# Impact of the COVID-19 Pandemic and Public Health Measures on Hepatitis C Treatment Initiation in Ontario, Canada

**DOI:** 10.3390/v18101090

**Published:** 2026-10-02

**Authors:** Yeva Sahakyan, Samantha S. M. Drover, William W. L. Wong, Zoë R. Greenwald, Alexander Kopp, Richard L. Morrow, Naveed Z. Janjua, Beate Sander

**Affiliations:** 1 Health Systems and Policy Research Collaborative Centre, University Health Network, Toronto, ON M5G 2C4, Canada; yeva.sahakyan@uhn.ca; 2 ICES, Toronto, ON M4N 3M5, Canada; samantha.drover@ices.on.ca (S.S.M.D.); zoe.greenwald@mail.utoronto.ca (Z.R.G.); alexander.kopp@ices.on.ca (A.K.); 3 School of Pharmacy, University of Waterloo, Kitchener, ON N2G 1C5, Canada; william.wl.wong@uwaterloo.ca; 4 Dalla Lana School of Public Health, University of Toronto, Toronto, ON M5T 3M7, Canada; 5 British Columbia Centre for Disease Control (BCCDC), Vancouver, BC V5Z 4R4, Canada; richard.morrow@bccdc.ca (R.L.M.); naveed.janjua@bccdc.ca (N.Z.J.); 6 School of Population and Public Health, University of British Columbia, Vancouver, BC V6T 1Z3, Canada; 7 Institute of Health Policy, Management, and Evaluation, University of Toronto, Toronto, ON M5T 3M6, Canada; 8 Public Health Ontario, Toronto, ON M5G 1M1, Canada

**Keywords:** hepatitis C, COVID-19, population-based cohort, administrative data, priority populations, interrupted time series, treatment rates

## Abstract

The impact of the COVID-19 pandemic and related public health measures on hepatitis C (HCV) treatment uptake in Ontario remains uncertain. We evaluated changes in HCV treatment initiation during the pandemic overall and among priority populations. We conducted a population-based study using Ontario health administrative data from January 2015 to December 2022. The primary outcome was HCV treatment initiation rate, defined as the first antiviral dispensation per 1000 eligible individuals. Interrupted time series analysis estimated changes during the pandemic relative to counterfactual projections based on pre-pandemic trends. We stratified analyses by birth cohort, immigration status, homelessness, people who inject drugs (PWID), and human immunodeficiency virus status. A sensitivity analysis restricted the pre-pandemic period to 2018 onward. Among 63,691 eligible individuals, treatment initiation increased from 2015, peaked in 2017, and declined thereafter, resulting in a flat pre-pandemic trend. Treatment initiation declined at pandemic onset and continued downward through 2022; however, neither level nor slope changes were statistically significant. Results were sensitive to pre-pandemic period specification: restricting analyses to 2018 onward showed a significant pre-pandemic decline, with observed pandemic-period rates exceeding counterfactual projections. Younger cohorts, PWID, and individuals experiencing homelessness showed heterogeneous trends. We found no clear evidence that the COVID-19 pandemic independently altered HCV treatment initiation trends in Ontario, although treatment initiation declined at pandemic onset and remained lower throughout the pandemic period.

## 1. Introduction

In Canada, chronic hepatitis C virus (HCV) infection remains a public health concern, affecting an estimated 5.6 per 1000 Canadians [1]. Direct-acting antivirals (DAAs) have transformed HCV care, achieving cure in over 90% of treated individuals [2,3]. In 2016, Canada endorsed the World Health Organization (WHO) targets to eliminate HCV by 2030, aiming for 90% of infections diagnosed, 80% of eligible individuals treated, and a 65% reduction in HCV-related mortality [4,5,6]. Ontario, home to nearly 40% of the Canadian population, has implemented initiatives in support of these goals, including risk-based screening, the introduction of point-of-care diagnostics, and public reimbursement of antivirals [7,8,9]. Since the introduction of all-oral DAAs in 2014, treatment uptake in Canada increased steadily, peaking in 2018 before beginning to decline in 2019 [7]. The COVID-19 pandemic and associated public health measures significantly strained national health systems, posing major challenges to achieving the WHO’s HCV elimination targets.

Ontario declared a state of emergency on 17 March 2020, and experienced one of the longest cumulative durations of emergency orders in Canada (214 days) [10,11,12]. This prolonged period of restrictions led to a more sustained disruption of non-essential health services, including routine and specialized HCV care, compared with other jurisdictions [13]. Consequently, Ontario observed sustained decreases in HCV testing rates that remained 41% below the expected levels in 2022 [14,15]. In contrast, in British Columbia, HCV testing and treatment declined sharply at the onset of the pandemic but recovered by the end of 2020 [16,17]. While the impact of COVID-19 on HCV testing in Ontario is documented, less is known about the downstream effect on HCV treatment initiation. An earlier study showed a nearly 50% reduction in HCV treatment dispensations during the first year of the pandemic [18], though the long-term consequences remain unknown.

Despite pre-pandemic elimination efforts, gaps in HCV testing and care persisted, especially among people who inject drugs (PWID), people experiencing homelessness, and those living with human immunodeficiency virus (HIV) [5,19]. Recent modeling studies showed that Ontario is not on track to meet WHO’s HCV elimination targets by 2030 [20,21]. Understanding the magnitude and duration of pandemic-related HCV treatment interruptions, and whether these disruptions widened existing gaps among priority populations, is essential for informing post-pandemic recovery strategies and future preparedness planning. This study aims to evaluate the impact of the COVID-19 pandemic and associated public health measures on HCV treatment rates overall and among priority populations in Ontario, Canada.

## 2. Materials and Methods

### 2.1. Study Design, Setting, and Population

This population-based cohort study estimated the impact of the COVID-19 pandemic on HCV treatment rates using health administrative databases. The cohort included all Ontario residents with a positive HCV antibody or HCV ribonucleic acid (RNA) or genotype test available in electronic health records during the pre-pandemic (1 January 2015 to 29 February 2020) or pandemic periods (1 March 2020 to 31 December 2022), with March 2020 classified as part of the pandemic period to approximate the timing of COVID-19 related disruptions. We selected 2015 as the start of the study period because it marked the initial introduction of DAA treatments to the Ontario public drug formulary. The index date was defined as the earliest positive test date, and the cohort assignment (pre-pandemic or pandemic) was based on this date. Individuals without a valid Ontario Health Insurance Plan (OHIP) number, those over 100 years of age, individuals with missing age or sex data, and those whose first test date was recorded on or after their date of death were excluded. This study follows the RECORD reporting guidelines for observational research [22].

### 2.2. Variable Definitions and Data Sources

We linked health administrative datasets held at ICES using unique encoded identifiers and analyzed the data at ICES. These datasets capture healthcare use for nearly all Ontario residents covered by the province’s publicly funded healthcare system. Table S1 summarizes the selected ICES datasets used in this study.

We collected data on HCV antibody, RNA and genotype test results from the Ontario Laboratories Information System (OLIS), a province-wide repository that captures orders from hospitals, commercial and public health laboratories. We used the Ontario Drug Benefit (ODB) database and drug identification numbers to obtain information on antiviral dispensations (Table S2). In Ontario, nearly 90% of DAAs are covered by the ODB [18,23].

Priority populations disproportionately affected by HCV infection included immigrants, people experiencing homelessness, people living with HIV, and PWID who also face differential access to healthcare services that may limit timely testing and treatment [1,5,24,25,26]. We collected immigration status from the Immigration, Refugees, and Citizenship Canada (IRCC) Permanent Resident Database, which holds records of individuals landed in Ontario and were granted permanent residency in Canada since 1985. We classified homelessness using a validated algorithm based on diagnostic and postal codes over a two-year lookback period (Table S3) [27,28]. HIV status followed a case definition of three or more HIV-related physician claims within three years [29]. We identified PWID status using a validated high-specificity algorithm capturing any indication of injecting behavior, including at least one hospitalisation or emergency department visit for drug dependence or a record of opioid agonist therapy (Table S4) [30].

We obtained demographic information from the Registered Persons Database (RPDB). Neighbourhood-level social marginalization was assessed using the Ontario Marginalization Index (ONMARG) quintiles for material resources, households and dwellings, age and labor force, and racialized and newcomer populations. Comorbidities were defined using the Johns Hopkins Adjusted Clinical Group^®^ (ACG) system and the Aggregated Diagnosis Groups (ADG) classification scheme applied to the 2-year period preceding the index date [31]. Cirrhosis, decompensated cirrhosis, hepatocellular carcinoma, and liver transplantation were identified using validated algorithms for Ontario administrative datasets (Tables S5–S8) [32].

### 2.3. Statistical Analysis

The primary outcome was the monthly rate of individuals initiating antiviral treatment, identified through the ODB database, calculated per 1000 individuals eligible for HCV treatment. Each individual was counted only once in the numerator, in the month of their first HCV treatment dispensation. The denominator included individuals eligible for HCV treatment, defined as those with a positive RNA or genotype test, and those with a positive HCV antibody test without a recorded RNA or genotype result (i.e., unavailable or inconclusive). Individuals who were antibody-positive with negative RNA or genotype testing and did not initiate treatment were excluded as this likely represents resolved HCV infections (spontaneous clearance or sustained virologic response to treatment). Each individual contributed to the denominator for one or more months until treatment initiation, death, or end of follow-up, whichever occurred first (e.g., an individual who tested positive in January 2022 and initiated antiviral therapy in April 2022 would be included in the denominator from January to April 2022).

To evaluate the impact of the pandemic on treatment rates, we used an interrupted time series approach in three steps. First, we fit a regression model with separate time trends for the pre-pandemic and pandemic phases, adjusted for autocorrelation. Next, we used the pre-pandemic trend to project counterfactual treatment rates for 2020–2022, assuming the pre-pandemic trend continued through the pandemic phase. Finally, we compared observed and counterfactual treatment rates by fitting a second model to a combined dataset of both actual and counterfactual rates, testing whether the two diverged overall and whether this divergence changed across 2020, 2021, and 2022. The standard errors for the observed-counterfactual differences were computed based on the Yule-Walker covariance matrix [33]. We stratified the analysis by sex, birth cohort, immigration status, homelessness, HIV, and PWID status to evaluate HCV treatment initiation rates among priority populations.

Because eligibility for publicly funded DAAs evolved over the pre-pandemic observation period and pan-genotypic regimens became broadly publicly funded in 2018 [7], we conducted Sensitivity Analysis 1 restricting the pre-pandemic segment to March 2018 onward, to minimize confounding due to policy-driven changes in treatment access that could influence pre-pandemic trends. In Sensitivity Analysis 2, we restricted the study population to individuals with a positive RNA or genotype test to assess the robustness of findings to a more restrictive definition of HCV infection. All analyses were conducted at ICES using SAS statistical software (SAS Enterprise Guide 8.3).

## 3. Results

### 3.1. Characteristics of the Study Cohort

A total of 87,532 individuals had at least one HCV antibody, RNA, or genotype test during the study period, of whom 63,691 were identified as eligible for HCV treatment (Figure S1). Among those eligible for treatment, compared to the pre-pandemic cohort (*n* = 52,313, 62.6% male), the pandemic cohort (*n* = 11,378, 61.1% male) was younger (mean age 48.0 vs. 49.1 years) and included a higher proportion of individuals born in 1975 or later (48.1% vs. 31.5%). Sociodemographic profiles including immigration status, residency, and quintile distributions for neighborhood income, household and dwelling, material resources, and racialized and newcomer populations were comparable across periods. The proportion of individuals experiencing homelessness was higher during the pandemic compared with the pre-pandemic period (9.8% vs. 3.7% for homelessness), while the proportion of PWID was similar between the periods. HIV positivity was recorded in 1.1% of individuals during the pandemic and 2.3% pre-pandemic. Advanced liver disease was identified in 8.0% of individuals during the pandemic versus 14.3% pre-pandemic. ADG scores were similar across periods (Table 1).

**Figure 1:**
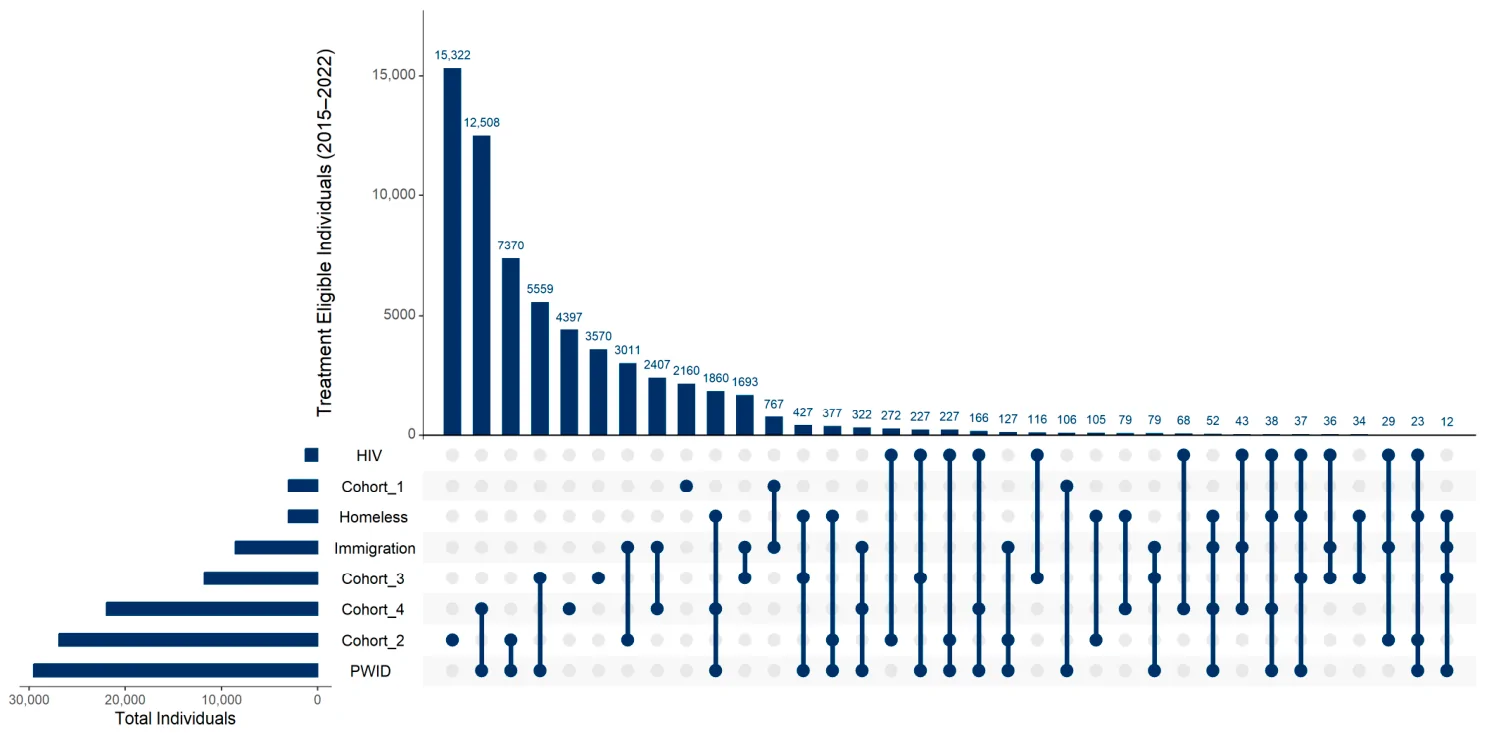
Intersectionality of priority populations among individuals eligible for HCV treatment, 2015–2022. Horizontal bars (**left**) show the number of individuals in each priority population. Vertical bars (**top**) show the number of individuals in each combination (intersection) of priority populations. Filled black circles indicate the populations included in each intersection, and connecting lines denote overlapping priority populations. Combinations with small cell counts were omitted. Birth cohort subcategories are defined as: <1945 (Cohort 1), 1945–1964 (Cohort 2), 1965–1974 (Cohort 3), and ≥1975 (Cohort 4); HIV: human immunodeficiency virus; PWID: people who inject drugs.

Among 63,691 treatment-eligible individuals (2015–2022), the most common intersecting combinations involved PWID with younger birth cohorts ≥1965 (*n* = 21,306) and immigration with birth cohort 1945–1964 (*n* = 3178). Smaller subgroups reflected co-occurring risk factors including HIV, homelessness across multiple birth cohorts (Figure 1).

### 3.2. HCV Treatment Rates in the Overall Cohort and Among Priority Populations

The overall median monthly number of individuals who initiated HCV treatment was 324 (interquartile range [IQR]: 207–383) during the pre-pandemic period and 208 (IQR: 186–225) during the pandemic period (Table S9). The absolute annual number of individuals initiating treatment increased from 2015, peaked in 2017 (Figure S2), then declined until 2020 and stabilized thereafter.

**Figure 2:**
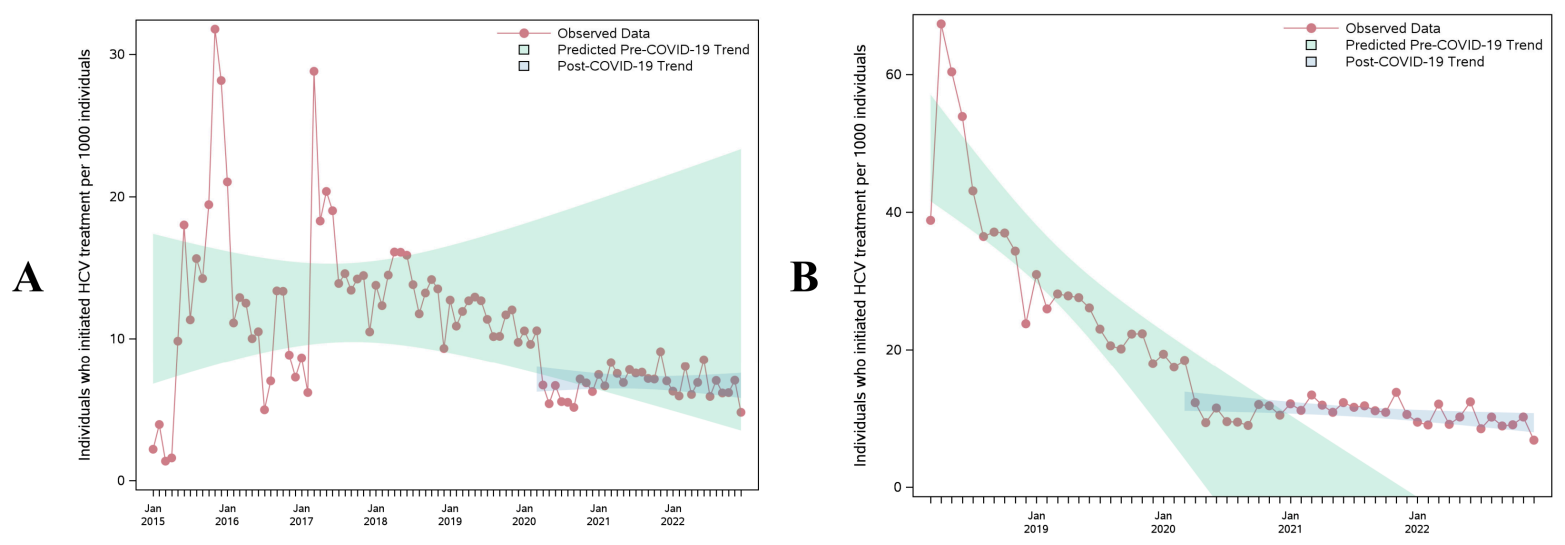
Observed and counterfactual HCV treatment initiation monthly rates with 95% confidence band for the main analysis (**A**), and Sensitivity Analysis 1 [2018–2022] (**B**). Rates were calculated per 1000 eligible individuals, defined as those with a positive RNA or genotype test, or those with a positive HCV antibody test without a RNA or genotype result (i.e., unavailable or inconclusive). HCV: hepatitis C virus; RNA: ribonucleic acid.

In the overall cohort, pre-pandemic monthly treatment initiation rates varied, but the modeled slope was roughly flat. At the onset of the COVID-19 pandemic treatment rates showed a non-significant immediate decline of 3.38 per 1000. During the pandemic, treatment rates continued to decline over time; however, these differences were not statistically significant (Figure 2, Table 2).

When pre-pandemic trends were extrapolated to project counterfactual estimates, observed treatment initiation rates were consistently lower than expected throughout 2020–2022. The absolute differences were −5.94 (95% CI: −6.70 to −5.18) in 2020 (March–December), −5.65 (95% CI: −6.34 to −4.95) in 2021, and −6.77 (95% CI: −7.47 to −6.08) in 2022 per 1000 eligible individuals (Table 3).

**Figure 3:**
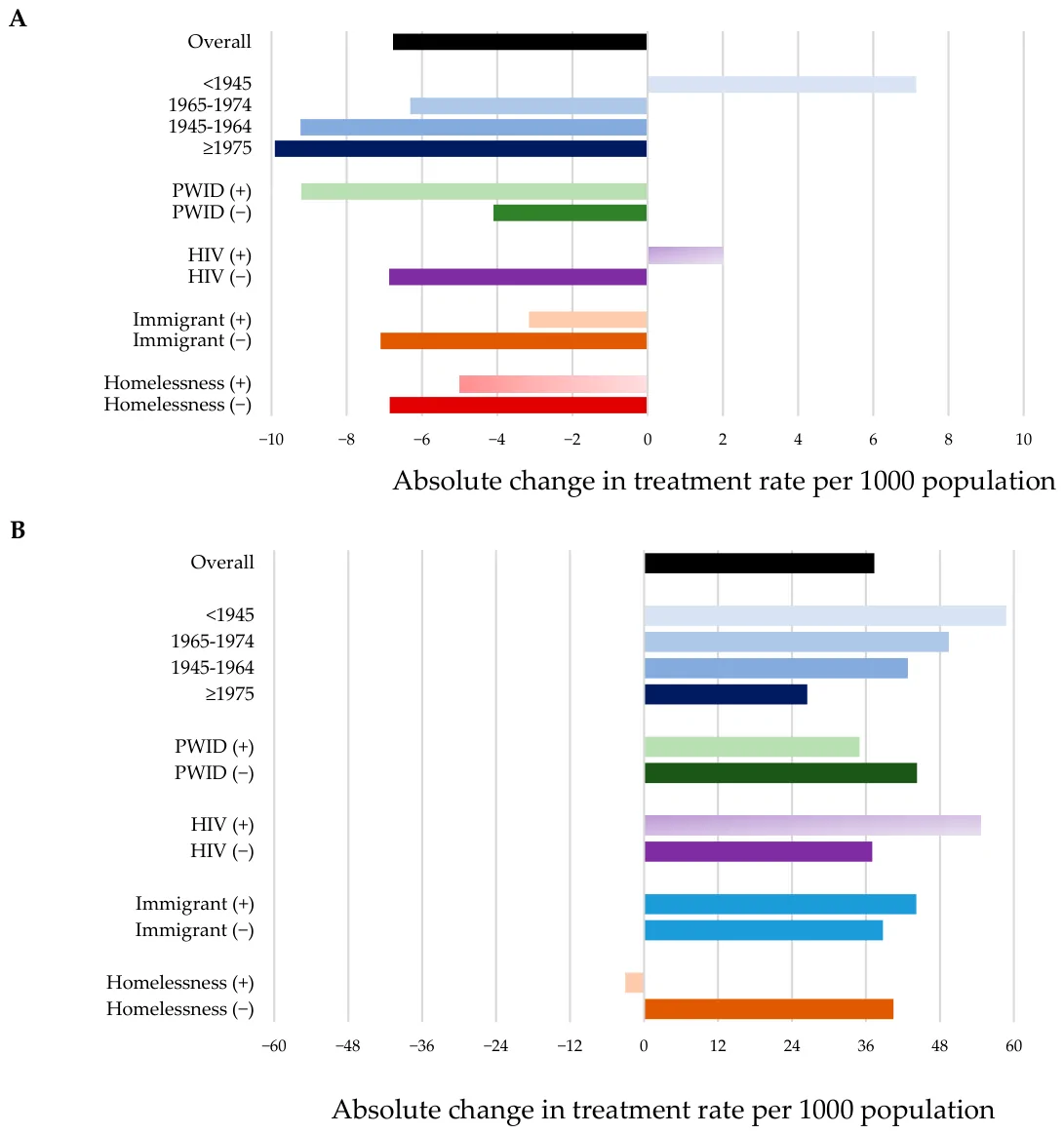
Absolute change in HCV treatment initiation rates in 2022 compared with pre-pandemic levels overall and by priority population, shown for the main analysis [2015–2022] (**A**), and Sensitivity Analysis 1 [2018–2022] (**B**). Rates were calculated per 1000 eligible individuals, defined as those with a positive RNA or genotype test, as well as those with a positive HCV antibody test but without a RNA or genotype result (i.e., unavailable or non-confirmatory). HCV: hepatitis C virus; HIV: human immunodeficiency virus; PWID: people who inject drugs; RNA: ribonucleic acid.

Several patterns emerged among priority populations. Trends by sex (Figure S3) and immigration status (Figure S4) were directionally consistent with those observed in the overall population, characterized by generally flat pre-pandemic slope, declines at pandemic onset, and negative post-pandemic trends, with neither the level change nor the post-pandemic trend reaching statistical significance. Individuals born before 1945 (Figure S5) and those living with HIV (Figure S6) exhibited declining pre-pandemic trends with no significant pandemic-related changes, resulting in observed rates that exceeded counterfactual projections in 2022 (Table 2 and Table 3, Figure 3). In contrast, younger birth cohorts (1965–1974 and ≥1975) showed increasing pre-pandemic trends followed by significant declines at pandemic onset, with no subsequent recovery (Figure S5). Among PWID, treatment rates increased pre-pandemic, declined at pandemic onset, and subsequently plateaued (Figure S7). Individuals experiencing homelessness showed increasing pre-pandemic trend and a pronounced decline at pandemic onset compared with other subgroups, with partial but incomplete recovery thereafter (Figure S8).

### 3.3. Sensitivity Analyses

Compared with the main analysis, restricting the pre-pandemic period to March 2018 onward resulted in a different trajectory of pre-pandemic treatment rates, consistent with peak DAA uptake. We observed a statistically significant downward trend prior to the pandemic (−1.57 per month per 1000 eligible individuals) (Table S10). Thus, when pre-pandemic trends were extrapolated to project counterfactual estimates, observed treatment initiation rates were consistently higher than expected throughout 2020–2022, overall and across all priority populations (Figure 2B and Figure 3B, Table S11). An exception was individuals experiencing homelessness, who exhibited no clear pre-pandemic trend but a pronounced decline at pandemic onset relative to other subgroups, followed by partial but incomplete recovery (Tables S10 and S11).

In Sensitivity Analysis 2, restricting the treatment-eligible population to individuals with a positive RNA or genotype test yielded results consistent with the main analysis (Tables S12 and S13).

## 4. Discussion

We evaluated the impact of the COVID-19 pandemic on HCV treatment initiation in Ontario, overall and among priority populations. Treatment initiation increased from 2015, peaked in 2017, and declined thereafter, consistent with changes in eligibility criteria for DAA reimbursement [7]. At pandemic onset, treatment rates declined, followed by a continued downward trend during 2020–2022; however, neither the level change nor the slope was statistically significant. Although observed rates were lower than counterfactual projections during 2020–2022 in the main analysis, these differences were sensitive to specification of pre-pandemic trends. In Sensitivity Analysis 1 restricting the pre-pandemic period to 2018 onward, we observed a significant pre-pandemic decline in treatment initiation, reflecting a peak in DAA uptake in 2018. This altered the direction of post-pandemic differences, such that observed treatment initiation rates were higher than counterfactual projections throughout 2020–2022. These findings suggest that observed post-2020 differences largely reflect underlying pre-pandemic treatment dynamics. Although treatment initiation levels appeared to decline at pandemic onset and remained lower throughout the pandemic period, the sensitivity of the counterfactual estimates to pre-pandemic trend specification limits our ability to attribute these differences to the pandemic.

The influence of pre-pandemic treatment dynamics was particularly evident among younger birth cohorts. In the main analysis, this cohort appeared to experience pandemic-related disruption in treatment initiation, which may be attributable to both the increasing pre-pandemic treatment trend and the higher representation of PWID within this group (Figure 1), who may be particularly vulnerable to disruptions in healthcare access and continuity of HCV care. However, the increasing pre-pandemic treatment trends observed in the main analysis should be interpreted in the context of evolving DAA reimbursement policies. Prior to the expansion of treatment eligibility in 2017, access was largely restricted to individuals with more advanced liver disease, a less common condition among younger individuals. Consequently, the apparent pre-pandemic increase in treatment initiation among younger cohorts may partly reflect treatment uptake following eligibility expansion rather than a true underlying increase in treatment demand. This interpretation is supported by Sensitivity Analysis 1, in which pre-pandemic trends among younger cohorts were declining rather than increasing.

A notable exception was individuals experiencing homelessness, whose findings were consistent across both the main and sensitivity analyses. In all analyses, treatment initiation declined significantly at pandemic onset, followed by a positive post-pandemic trend. However, observed treatment rates remained below counterfactual projections through 2022, suggesting only partial recovery. The only difference was the pre-pandemic trend, which was positive in the main analysis but essentially flat in Sensitivity Analysis 1. Individuals experiencing homelessness may have been particularly vulnerable to disruptions in healthcare delivery, including reduced outpatient capacity, redeployment of clinical staff, and restrictions on in-person care [11,12,34], given their reliance on outreach-based and socially supportive models of care. The substantial overlap between homelessness and PWID may have further contributed to challenges in maintaining engagement in HCV care during this period [25]. While the underlying mechanisms cannot be directly assessed in this study, the consistency of findings across analytic approaches suggests that individuals experiencing homelessness may have experienced greater challenges in accessing HCV treatment than other priority populations.

Earlier studies have documented pandemic-related changes in HCV treatment in Canada and worldwide [17,35,36,37]. In British Columbia, a study with a follow-up extending to 2022, found a short-term 26% decline in HCV treatment initiation in 2020, which was most pronounced among people born between 1965 and 1974 and PWID, and rebounded after 2020 across all subgroups [17]. Similarly, an earlier Ontario study with shorter follow-up, reported a nearly 50% reduction in DAA prescriptions from 2018 to 2020 [18]. However, unlike these studies, we did not find statistically robust evidence of an independent pandemic-related change in treatment initiation after accounting for pre-pandemic trends. Differences likely reflect variation in follow-up and pre-pandemic trend specifications, as well as differences in healthcare policies, care pathways, and pandemic restrictions across jurisdictions. Importantly, the magnitude and duration of the disruption observed in Ontario differed from those reported in other jurisdictions, highlighting the importance of jurisdiction-specific policy and healthcare contexts when assessing the pandemic’s impact on HCV care.

This study has several limitations. First, changes in DAA reimbursement eligibility influenced estimates of pre-pandemic trends, with publicly funded access expanding following formulary listing in 2015, broader limited-use coverage in 2017, and the introduction of pan-genotypic DAAs in 2018 [7]. Because the pandemic coincided with an ongoing decline in HCV treatment initiation, distinguishing pandemic-related effects from underlying trends was challenging, and counterfactual estimates were sensitive to the pre-pandemic period selected. Second, hepatitis C treatment initiation may be under-ascertained in our analysis, as Ontario does not have universal drug coverage [38] and some prescriptions may not be captured under public coverage. Previous analysis, however, estimated that approximately 88% of DAA prescriptions in Ontario were publicly funded [23], indicating that the majority of treatment initiations are likely represented. Third, there is potential misclassification of priority populations due to limitations in administrative data coding, particularly for individuals experiencing homelessness. Although coding improved after 2018 with mandatory documentation of housing status in hospital encounters, estimates of the pandemic effect and trend were consistent across main and sensitivity analyses, suggesting minimal impact on the observed results [28]. Finally, subgroup analyses were based on smaller sample sizes than the overall population and were not formally adjusted for multiple comparisons across priority populations, therefore, they should be interpreted cautiously.

This study has several strengths. First, we used population-based administrative health data capturing nearly all Ontario residents eligible for publicly funded healthcare, providing a comprehensive assessment of HCV treatment initiation across the province and among priority populations. Second, the interrupted time series design with counterfactual modelling, supported by sensitivity analyses using alternative pre-pandemic periods, and a more restrictive treatment eligible population enabled a transparent assessment of how pre-pandemic trend specification influences inferred post-pandemic effects. Third, by examining treatment initiation rates among individuals eligible for treatment rather than crude treatment counts, we accounted for changes in the size of the treatment-eligible population over time, providing a more meaningful measure of engagement in HCV care. However, pandemic-related reductions in HCV testing could have reduced the identification of newly eligible individuals, thereby affecting both the denominator and the number subsequently initiating treatment; the net effect on treatment initiation rates is therefore uncertain. Finally, when considered alongside our previous work showing decline in HCV testing during the pandemic in Ontario [15], these findings provide insight into pandemic-related disruptions across multiple stages of the HCV care cascade and their implications for HCV elimination efforts. Because treatment initiation was assessed among individuals who were tested, changes in testing volume and practices during the pandemic may have influenced the characteristics of the population entering the treatment cascade.

## 5. Conclusions

Overall, we found no clear evidence that the COVID-19 pandemic independently changed HCV treatment initiation trends in Ontario, although treatment initiation levels appeared to decline at pandemic onset and remained lower throughout the pandemic period. Our findings highlight the importance of ongoing monitoring of HCV treatment patterns, particularly among individuals experiencing homelessness, to support continued progress toward HCV elimination.

## Figures and Tables

**Table 1:** Characteristics of the study cohort deemed eligible for antiviral treatment.

Characteristics	Pre-Pandemic 1 January 2015–29 February 2020 *N* = 52,313	Pandemic 1 March 2020–31 December 2022 *N* = 11,378
**Age at index, years, mean (sd)**	49.1 (14.9)	48.0 (16.4)
**Birth year, *n* (%)**		
<1945	2596 (5.0)	463 (4.1)
1945–1964	23,308 (44.6)	3567 (31.3)
1965–1974	9928 (19.0)	1871 (16.4)
≥1975	16,481 (31.5)	5477 (48.1)
**Male sex, *n* (%)**	32,735 (62.6)	6952 (61.1)
**Immigrant status, *n* (%)**	7000 (13.4)	1945 (17.1)
**Rural, *n* (%)**	5440 (10.5)	1422 (12.7)
**Neighborhood income quintile, *n* (%)**		
1st quintile (lowest)	20,215 (39.0)	4226 (37.6)
2nd quintile	11,616 (22.4)	2447 (21.5)
3rd quintile	8407 (16.2)	1890 (16.8)
4th quintile	6353 (12.2)	1453 (12.8)
5th quintile	5241 (10.1)	1220 (10.7)
**Household and dwellings quintile, *n* (%)**		
1st quintile (lowest)	5554 (10.9)	1310 (12.0)
2nd quintile	6025 (11.9)	1374 (12.6)
3rd quintile	7609 (14.9)	1710 (15.6)
4th quintile	11,272 (22.2)	2391 (21.9)
5th quintile	20,307 (40.0)	4142 (37.9)
**Material resources quintile, *n* (%)**		
1st quintile (lowest)	5866 (11.5)	1219 (11.1)
2nd quintile	7069 (13.9)	1690 (15.5)
3rd quintile	8215 (16.2)	1868 (17.1)
4th quintile	10,647 (21.0)	2284 (20.9)
5th quintile	18,970 (37.4)	3866 (35.4)
**Racialized and newcomer populations quintile, *n* (%)**		
1st quintile (lowest)	9181 (18.1)	2069 (19.0)
2nd quintile	9348 (18.5)	1928 (17.6)
3rd quintile	9724 (19.2)	2086 (19.1)
4th quintile	10,420 (20.5)	2188 (20.0)
5th quintile	12,094 (23.8)	2656 (24.3)
**Homelessness, *n* (%)**	1952 (3.7)	1113 (9.8)
**PWID ever, *n* (%)**	24,294 (46.4)	5273 (46.3)
**HIV positivity, *n* (%)**	1194 (2.3)	123 (1.1)
**ADG score, mean (sd)**	17.9 (14.3)	17.3 (14.5)
**ADG categories, *n* (%)**		
0–3	9859 (18.8)	2617 (23.0)
4–8	21,988 (42.0)	4751 (41.8)
9–10	7513 (14.4)	1471 (12.9)
≥11	12,953 (24.8)	2539 (22.3)
**Advanced liver disease**		
Any advanced liver disease, *n* (%)	7484 (14.3)	910 (8.0)
Cirrhosis, *n* (%)	7295 (13.9)	866 (7.6)
Decompensated cirrhosis, *n* (%)	1438 (2.7)	192 (1.7)
HCC, *n* (%)	645 (1.2)	128 (1.1)
Liver transplant recipients, *n* (%)	338 (0.6)	25 (0.2)

This table includes individuals eligible for treatment defined as those with a positive RNA or genotype test, as well as those with a positive HCV antibody test without a recorded RNA or genotype result (i.e., unavailable or inconclusive). The characteristics are as of the index date, which is the earliest of the antibody or RNA or genotype test date. ADG: aggregated diagnostic groups; HCC: hepatocellular carcinoma; HIV: human immunodeficiency virus; N: number of observations. PWID: people who inject drugs; RNA: ribonucleic acid; sd: standard deviation.

**Table 2:** Treatment initiation rates, the result of interrupted time series analysis.

Model Parameter	Estimate	SE	*p*-Value
**Overall** Intercept Pre-pandemic treatment rate slope, monthly	12.433 −0.002	2.14 0.06	0.000 0.977
Study period (pandemic, ref: pre-pandemic)	−3.381	2.99	0.260
Pandemic treatment rate slope, monthly	−0.094	0.13	0.478
Difference in slopes (pandemic vs. pre-pandemic)	−0.092	0.15	0.544
**Female**			
Intercept	11.281	2.17	0.000
Pre-pandemic treatment rate slope, monthly	0.007	0.06	0.906
Study period (pandemic, ref: pre-pandemic)	−3.223	2.99	0.284
Pandemic treatment rate slope, monthly	−0.108	0.13	0.420
Difference in slopes (pandemic vs. pre-pandemic)	−0.114	0.15	0.457
**Male**			
Intercept	13.15	2.12	0.000
Pre-pandemic treatment rate slope, monthly	−0.006	0.06	0.918
Study period (pandemic, ref: pre-pandemic)	−3.669	3.04	0.230
Pandemic treatment rate slope, monthly	−0.078	0.13	0.559
Difference in slopes (pandemic vs. pre-pandemic)	−0.072	0.15	0.635
**<1945**			
Intercept	24.726	3.23	0.000
Pre-pandemic treatment rate slope, monthly	−0.321	0.09	0.000
Study period (pandemic, ref: pre-pandemic)	−0.519	4.72	0.913
Pandemic treatment rate slope, monthly	−0.045	0.20	0.821
Difference in slopes (pandemic vs. pre-pandemic)	0.276	0.22	0.220
**1945–1964**			
Intercept	17.777	2.99	0.000
Pre-pandemic treatment rate slope, monthly	−0.088	0.08	0.276
Study period (pandemic, ref: pre-pandemic)	−4.212	4.06	0.302
Pandemic treatment rate slope, monthly	−0.129	0.18	0.482
Difference in slopes (pandemic vs. pre-pandemic)	−0.041	0.21	0.847
**1965–1974**			
Intercept	10.570	1.68	0.000
Pre-pandemic treatment rate slope, monthly	0.052	0.05	0.262
Study period (pandemic, ref: pre-pandemic)	−5.016	2.57	0.054
Pandemic treatment rate slope, monthly	−0.075	0.11	0.493
Difference in slopes (pandemic vs. pre-pandemic)	−0.126	0.12	0.299
**≥1975**			
Intercept	2.958	1.10	0.009
Pre-pandemic treatment rate slope, monthly	0.165	0.03	0.000
Study period (pandemic, ref: pre-pandemic)	−3.559	1.61	0.030
Pandemic treatment rate slope, monthly	−0.046	0.07	0.502
Difference in slopes (pandemic vs. pre-pandemic)	−0.211	0.08	0.007
**PWID (ever): YES**			
Intercept	9.510	1.70	0.000
Pre-pandemic treatment rate slope, monthly	0.117	0.05	0.013
Study period (pandemic, ref: pre-pandemic)	−4.913	2.58	0.060
Pandemic treatment rate slope, monthly	−0.011	0.11	0.920
Difference in slopes (pandemic vs. pre-pandemic)	−0.128	0.12	0.297
**PWID (ever): NO**			
Intercept	14.604	2.55	0.000
Pre-pandemic treatment rate slope, monthly	−0.093	0.07	0.178
Study period (pandemic, ref: pre-pandemic)	−2.725	3.38	0.423
Pandemic treatment rate slope, monthly	−0.114	0.15	0.461
Difference in slopes (pandemic vs. pre-pandemic)	−0.021	0.18	0.905
**Immigrant: YES**			
Intercept	11.644	2.08	0.000
Pre-pandemic treatment rate slope, monthly	−0.079	0.06	0.164
Study period (pandemic, ref: pre-pandemic)	−2.576	2.95	0.384
Pandemic treatment rate slope, monthly	−0.083	0.13	0.520
Difference in slopes (pandemic vs. pre-pandemic)	−0.005	0.15	0.975
**Immigrant: NO**			
Intercept	12.626	2.12	0.000
Pre-pandemic treatment rate slope, monthly	0.011	0.06	0.846
Study period (pandemic, ref: pre-pandemic)	−3.655	3.01	0.227
Pandemic treatment rate slope, monthly	−0.083	0.13	0.531
Difference in slopes (pandemic vs. pre-pandemic)	−0.094	0.15	0.534
**HIV: YES**			
Intercept	22.374	3.31	0.000
Pre-pandemic treatment rate slope, monthly	−0.195	0.09	0.030
Study period (pandemic, ref: pre-pandemic)	−3.824	4.85	0.433
Pandemic treatment rate slope, monthly	0.009	0.20	0.966
Difference in slopes (pandemic vs. pre-pandemic)	0.204	0.22	0.357
**HIV: NO**			
Intercept	12.281	2.12	0.000
Pre-pandemic treatment rate slope, monthly	0.001	0.06	0.987
Study period (pandemic, ref: pre-pandemic)	−3.358	2.94	0.257
Pandemic treatment rate slope, monthly	−0.095	0.13	0.467
Difference in slopes (pandemic vs. pre-pandemic)	−0.096	0.15	0.523
**Homelessness: YES**			
Intercept	3.834	1.76	0.033
Pre-pandemic treatment rate slope, monthly	0.226	0.05	0.000
Study period (pandemic, ref: pre-pandemic)	−5.057	2.37	0.035
Pandemic treatment rate slope, monthly	0.248	0.10	0.011
Difference in slopes (pandemic vs. pre-pandemic)	0.022	0.11	0.838
**Homelessness: NO**			
Intercept	12.739	2.17	0.000
Pre-pandemic treatment rate slope, monthly	−0.011	0.06	0.853
Study period (pandemic, ref: pre-pandemic)	−3.310	3.03	0.278
Pandemic treatment rate slope, monthly	−0.108	0.13	0.421
Difference in slopes (pandemic vs. pre-pandemic)	−0.097	0.15	0.528

HIV: human immunodeficiency virus; PWID: people who inject drugs; ref: reference; SE: standard error.

**Table 3:** Observed vs. counterfactual treatment initiation rates per 1000 eligible individuals, overall and stratified by priority groups.

Population	Absolute Difference (95% CI)		
	May–December 2020	January–December 2021	January–December 2022
**Overall**	−5.94 (−6.70; −5.18)	−5.65 (−6.34; −4.95)	−6.77 (−7.47; −6.08)
**Sex**			
Female	−5.86 (−6.56; −5.17)	−5.89 (−6.53; −5.26)	−7.29 (−7.92; −6.65)
Male	−6.02 (−6.89; −5.14)	−5.48 (−6.28; −4.69)	−6.40 (−7.20; −5.61)
**Birth cohort**			
<1945	0.21 (−0.85; 1.26)	3.60 (2.57; 4.63)	7.14 (6.13; 8.14)
1945–1964	−6.69 (−7.36; −6.03)	−5.89 (−6.50; −5.28)	−6.31 (−6.92; −5.70)
1965–1974	−7.19 (−8.10; −6.27)	−7.31 (−8.14; −6.47)	−9.23 (−10.07; −8.40)
≥1975	−6.15 (−7.27; −5.03)	−7.11 (−8.13; −6.08)	−9.91 (−10.94; −8.89)
**PWID status**			
Yes	−7.70 (−8.93; −6.48)	−7.27 (−8.39; −6.15)	−9.21 (−10.33; −8.09)
No	−4.45 (−4.91; −3.98)	−3.99 (−4.42; −3.57)	−4.07 (−4.50; −3.65)
**Immigration status**			
Yes	−3.30 (−3.83; −2.77)	−3.06 (−3.55; −2.58)	−3.15 (−3.63; −2.66)
No	−6.35 (−7.20; −5.50)	−5.92 (−6.69; −5.14)	−7.10 (−7.88; −6.33)
**HIV status**			
HIV (+)	−3.47 (−5.03; −1.92)	−1.71 (−3.12; −0.29)	1.99 (0.58; 3.40)
HIV (−)	−5.95 (−6.72; −5.18)	−5.67 (−6.37; −4.97)	−6.87 (−7.57; −6.17)
**Homelessness status**			
Yes	−6.87 (−8.93; −4.81)	−3.01 (−4.88; −1.14)	−4.98 (−6.85; −3.11)
No	−5.83 (−6.61; −5.05)	−5.76 (−6.48; −5.04)	−6.86 (−7.58; −6.14)

CI: Confidence interval; HIV: human immunodeficiency virus; PWID: people who inject drugs.

## Data Availability

The dataset from this study is held securely in coded form at ICES. While legal data sharing agreements between ICES and data providers (e.g., healthcare organizations and government) prohibit ICES from making the dataset publicly available, access may be granted to those who meet pre-specified criteria for confidential access, available at https://www.ices.on.ca/DAS (email: das@ices.on.ca), accessed on 1 October 2026. The full dataset creation plan and underlying analytic code are available from the authors upon request, understanding that the computer programs may rely upon coding templates or macros that are unique to ICES and are therefore either inaccessible or may require modification.

## Supplementary Materials

The following supporting information can be downloaded at: https://www.mdpi.com/article/10.3390/v18101090/s1, Table S1: Selected ICES datasets used in this study; Table S2: HCV therapy and corresponding DINs; Table S3: Case definition of history of homelessness, considering a two-year lookback period; Table S4: Algorithm to identify people who inject drugs; Table S5: Codes to identify cirrhosis; Table S6: Codes to identify decompensated cirrhosis; Table S7: Codes to identify hepatocellular carcinoma; Table S8: Codes to identify liver transplantation; Table S9: Number of individuals per month initiating HCV treatment, overall and by priority groups; Table S10: Sensitivity Analysis 1 restricting study period to 2018–2022: Treatment initiation rates, the result of interrupted time series analysis; Table S11: Sensitivity Analysis 1 restricting study period to 2018–2022: Observed vs. Counterfactual treatment initiation rates per 1000 eligible individuals, overall and stratified by priority groups; Table S12: Sensitivity Analysis 2 restricting treatment-eligible population to individuals with a positive RNA or genotype test: Treatment initiation rates, the result of interrupted time series analysis; Table S13: Sensitivity Analysis 2 restricting treatment-eligible population to individuals with a positive RNA or genotype test: Observed vs. Counterfactual treatment initiation rates per 1000 eligible individuals, overall and stratified by priority groups; Figure S1: Study flowchart; Figure S2: Number of treatment eligible, treatment initiated individuals and treatment initiation rates based on ODB records for the 2015–2022 and 2018–2022 cohorts; Figure S3: Observed and counterfactual HCV treatment initiation rates by sex; Figure S4: Observed and counterfactual HCV treatment initiation rates by immigration status; Figure S5: Observed and counterfactual HCV treatment initiation rates by birth cohort; Figure S6: Observed and counterfactual HCV treatment initiation rates by HIV status; Figure S7: Observed and counterfactual HCV treatment initiation rates by PWID status; Figure S8: Observed and counterfactual HCV treatment initiation rates by homelessness status.

## Abbreviations

The following abbreviations are used in this manuscript:

DAA	Direct-acting antiviral
WHO	World Health Organization
PWID	People who inject drugs
HIV	Human immunodeficiency virus
OHIP	Ontario Health Insurance Plan
OLIS	Ontario Laboratories Information System
ODB	Ontario Drug Benefit
IRCC	Immigration, Refugees, and Citizenship Canada
RPD	Registered persons database
OMI	Ontario Marginalized Index
ACG	Adjusted Clinical Group
ADG	Aggregated diagnosis group
HCC	Hepatocellular carcinoma
RNA	Ribonucleic acid
