# Supplementary material for: Impact of the COVID-19 Pandemic and Public Health Measures on Hepatitis C Treatment Initiation in Ontario, Canada
Source: Viruses. 2026 Oct 2;18(10):1090. doi: 10.3390/v18101090 (PMC13709599; doi:10.3390/v18101090)
Supplement: Supplementary file 1 [file viruses-18-01090-s001.zip › viruses-4519843-supplementary.pdf]

THE IMPACT OF COVID-19 AND RELATED PUBLIC HEALTH MEASURES ON HEPATITIS C TREATMENT IN ONTARIO, CANADA

SUPPLEMENT

Table S1. Selected ICES datasets used in this study. 2

Table S2. HCV therapy and corresponding DINs. 3

Table S3. Case definition of history of homelessness, considering a two-year lookback period 4

Table S4. Algorithm to identify people who inject drugs 5

Table S5. Codes to identify cirrhosis 9

Table S6. Codes to identify decompensated cirrhosis. 10

Table S7. Codes to identify hepatocellular carcinoma 12

Table S8. Codes used to identify liver transplantation 13

Table S9. Number of individuals per month initiating HCV treatment, overall and by priority groups. 14

Table S10. Sensitivity Analysis 1 restricting study period to 2018-2022: Treatment initiation rates, the result of interrupted time series analysis. 15

Table S11. Sensitivity Analysis 1 restricting study period to 2018-2022: Observed vs. Counterfactual treatment initiation rates per 1,000 eligible individuals, overall and stratified by priority groups. 18

Table S12. Sensitivity Analysis 2 restricting the treatment-eligible population to individuals with a positive RNA or genotype test: Treatment initiation rates, the result of interrupted time series analysis. 19

Table S13. Sensitivity Analysis 2 restricting the treatment-eligible population to individuals with a positive RNA or genotype test: Observed vs. Counterfactual treatment initiation rates per 1,000 eligible individuals, overall and stratified by priority groups. 22

Figure S1. Study flowchart. 23

Figure S2. Number of treatment eligible, treatment initiated individuals and treatment initiation rates based on ODB records for the 2015-2022 and 2018-2022 cohorts. 24

Figure S3. Observed and counterfactual HCV treatment initiation rates by sex. 25

Figure S4. Observed and counterfactual HCV treatment initiation rates by immigration status. 26

Figure S5. Observed and counterfactual HCV treatment initiation rates by birth cohort. 27

Figure S6. Observed and counterfactual HCV treatment initiation rates by HIV status. 29

Figure S7. Observed and counterfactual HCV treatment initiation rates by PWID status. 30

Figure S8. Observed and counterfactual HCV treatment initiation rates by homelessness status. 31

References 32

Table S1. Selected ICES datasets used in this study.

List of datasets	Full name	Description
ACG	The Johns Hopkins ACG® System Version 10	Used in combination with OHIP, DAD, NACRS, and SDS to calculate Adjusted Clinical Groups (ACGs), Resource Utilization Band (RUB), and frailty
CIC/IRCC	Immigration Refugees and Citizenship Canada, Permanent Residents database	Used to determine immigration status
CORR	Canadian Organ Replacement Registry	Used to identify individuals with liver transplantation
DAD	Discharge Abstract Database	Used to identify priority populations
DIN	The DRUGLIST file (DIN: Drug Identification Numbers)	Used with ODB to identify priority populations and to identify hepatitis C treatments
HIV	Human immunodeficiency virus	Used to determine HIV status
NACRS	National Ambulatory Care Reporting System	Used to identify priority populations
NMS	Narcotics Monitoring System	Used to identify priority populations
OCR	Ontario Cancer Registry	Used to identify individuals with hepatocellular carcinoma
ODB	Ontario Drug Benefit Claims	Used to identify priority populations and to identify hepatitis C treatments
OHIP	Ontario Health Insurance Plan Claims Database	Used to determine eligibility, date of last contact with health care system
OLIS	Ontario Laboratory Information System	Used to determine those with hepatitis C testing
OMHRS	Ontario Mental Health Reporting System	Used to identify priority populations
ON-MARG	Ontario Marginalization Index	Used to create the Age and labour force quintiles, Material resource quintiles, Racialized and newcomer populations quintile, and Households and dwellings quintile
PCCF	Postal Code Conversion File	Used to determine postal code for Ontario residency
POP	Ontario Population Estimates and Projections	Used to estimate the denominators for testing rates
RPDB	Registered Persons Database	Demographics information on birth date, sex, and death date<br>Used to estimate the denominators for testing rates
SDS	Same Day Surgery Database	Used to identify priority populations

Source: For more information, please check https://datadictionary.ices.on.ca/Applications/DataDictionary/Default.aspx

Table S2. HCV therapy and corresponding DINs.

<table>
<tr><td>HCV treatment type</td><td>DIN</td><td>Drug name</td><td>Brand name</td></tr>
<tr><td rowspan="3">Interferon-treatments</td><td>02223406
02231651
02238674
02238675
02240693
02240694
02240695
02239730
02241159</td><td>INTERFERON</td><td>INTRON-A</td></tr>
<tr><td>02242966
02242967
02242968
02242969
02248077
02248078</td><td>PEGINTERFERON</td><td>PEGASYS</td></tr>
<tr><td>02246026
02246027
02246028
02246029
02246030
02253410
02253429
02254573
02254581
02254603
02254638
02254646
09854791</td><td>PEGINTERFERON, RIBAVIRIN</td><td>PEGETRON;
PEGETRON REDIPEN</td></tr>
<tr><td rowspan="4">First-generation DAAs (Interferon-based)</td><td>02370816</td><td>BOCEPREVIR</td><td>VICTRELIS</td></tr>
<tr><td>02371464
02371456
02371472
02371448
09857396</td><td>BOCEPREVIR & PEGINTERFERON ALFA 2B & RIBAVIRIN</td><td>VICTRELIS TRIPLE</td></tr>
<tr><td>02371553</td><td>TELAPREVIR</td><td>INCIVEK</td></tr>
<tr><td>02416441</td><td>SIMEPREVIR</td><td>GALEXOS</td></tr>
<tr><td rowspan="10">All-oral DAAs</td><td>02418355</td><td>SOFOSBUVIR</td><td>SOVALDI</td></tr>
<tr><td>02432226</td><td>LEDIPASVIR & SOFOSBUVIR</td><td>HARVONI</td></tr>
<tr><td>02436027</td><td>DASABUVIR & OMBITASVIR & PARITAPREVIR & RITONAVIR</td><td>HOLKIRA PAK</td></tr>
<tr><td>02447711</td><td>OMBITASVIR, PARITAPREVIR, RITONAVIR</td><td>TECHNIVIE</td></tr>
<tr><td>02452294</td><td>ASUNAPREVIR</td><td>SUNVEPRA</td></tr>
<tr><td>02451131</td><td>ELBASVIR & GRAZOPREVIR</td><td>ZEPATIER</td></tr>
<tr><td>02456370</td><td>SOFOSBUVIR & VELPATASVIR</td><td>EPCLUSA</td></tr>
<tr><td>02444755
02444747</td><td>DACLATASVIR HCL</td><td>DAKLINZA</td></tr>
<tr><td>02467542</td><td>SOFOSBUVIR & VELPATASVIR & VOXILAPREVIR</td><td>VOSEVI</td></tr>
<tr><td>02467550</td><td>GLECAPREVIR & PIBRENTASVIR</td><td>MAVIRET</td></tr>
</table>

Table S3. Case definition of history of homelessness, considering a two-year lookback period (1, 2).

Database	Variable name	Indicator value	Description
**DAD**	DX10CODE1 to DXCODE25	"Z590" or "Z591"	ICD-10 Diagnostic Codes for "Homelessness" and "Inadequate housing"
	CMGDIAG	"Z590" or "Z591"	ICD-10 Diagnostic Codes for "Homelessness" and "Inadequate housing"
	PSTLCODE	"XX"; list of postal codes uniquely identifying homeless shelters	Used to indicate transient/homeless patients
**NACRS**	DX10CODE1 to DX10CODE10	"Z590" or "Z591"	ICD-10 Diagnostic Codes for "Homelessness" and "Inadequate housing"
	RESTYEP	"3" or "4"	Residence Type = "Homeless" or "Shelter"
	PSTLCODE	"XX"; uniquely identifying shelter code	Used to indicate transient/homeless patients
**OMHRS**	PREDX10CODE to PREDX10CODE11	"Z590" or "Z591"	ICD-10 Diagnostic Codes for "Homelessness" and "Inadequate housing"
	POSTDX10CODE1 to POSTDX10CODE24	"Z590" or "Z591"	ICD-10 Diagnostic Codes for "Homelessness" and "Inadequate housing"
	PRIOR_RESIDENCE	"6"	Prior residental status = "Homeless (with or without shelter)"
	USUAL_RESIDENCE	"8"	Usual residental status = "Homeless (with or without shelter)"
	ADMITFROM	"8"	Admitted from = "Homeless (with or without shelter)"
	DISCHLIVING	"8"	Living arrangemenet at discharge = "Homeless (with or without shelter)"
	PSTLCODE	"XX"; uniquely identifying shelter code	Used to indicate transient/homeless patients
**RPDB PSTLYEAR**		uniquely identifying shelter code	Used to indicate transient/homeless patients

Table S4. Algorithm to identify people who inject drugs (3).

Variable	Data source	Code type	Codes	Description
**Physician billing codes for drug use**				
**Drug dependence**	OHIP	DXCODE	304	Drug dependence
	OHIP	FEECODE	A680	Substance use – initial assessment
	OHIP	FEECODE	C680	Substance use – initial assessment
	OHIP	FEECODE	K680	Substance abuse – extended assessment
	OHIP	FEECODE	A957	Focused Practice Assessment – Addiction medicine
**Emergency department and hospitalization codes for drug use (DAD, NACRS, OMHRS)**				
**Opioid use**	DAD, NACRS	ICD-9	3040	Drug dependence: Opioid type dependence
	DAD, NACRS	ICD-9	3047	Drug dependence: Combinations of opioid type drug with any other
	DAD, NACRS	ICD-9	3055	Nondependent abuse of drugs: Opioid abuse
	DAD, NACRS	ICD-9	9650	Poisoning by analgesics, antipyretics, and antirheumatics: opiates and related narcotics
	DAD, NACRS	ICD-9	E8500	Accidental poisoning by heroin
	DAD, NACRS	ICD-10	F11	Mental and behavioural disorders due to use of opioids
	DAD, NACRS	ICD-10	R781	Finding of opiate drug in blood
	DAD, NACRS	ICD-10	T400	Poisoning by Opium
	DAD, NACRS	ICD-10	T401	Poisoning by Heroin
	DAD, NACRS	ICD-10	T402	Poisoning by Other opioids
	DAD, NACRS	ICD-10	T403	Poisoning by Methadone
	DAD, NACRS	ICD-10	T404	Poisoning by Other synthetic narcotics
	DAD, NACRS	ICD-10	T406	Poisoning by Other and unspecified narcotics
	OHMRS	DSM-IV	30400	Opioid Dependence
	OHMRS	DSM-IV	30550	Opioid abuse
**Cocaine use**	DAD, NACRS	ICD-9	3042	Drug dependence: Cocaine dependence
	DAD, NACRS	ICD-9	3056	Nondependent abuse of drugs: Cocaine abuse
	DAD, NACRS	ICD-9	970	Poisoning by central nervous system stimulants
	DAD, NACRS	ICD-10	F14	Mental and behavioural disorders due to use of cocaine
	DAD, NACRS	ICD-10	T405	Poisoning by: Cocaine
	DAD, NACRS	ICD-10	R782	Finding of cocaine in blood
	OHMRS	DSM-IV	30420	Cocaine dependence
	OHMRS	DSM-IV	30560	Cocaine abuse
**Amphetamines**	DAD, NACRS	ICD-9	3044	Drug dependence: Amphetamine and other psychostimulant dependence

Variable	Data source	Code type	Codes	Description
	DAD, NACRS	ICD-9	3057	Nondependent abuse of drugs: Amphetamine or related acting sympathomimetic abuse
	DAD, NACRS	ICD-9	9697	Poisoning by Psychostimulants
	DAD, NACRS	ICD-10	F15	Mental and behavioural disorders due to use of other stimulants, including caffeine
	DAD, NACRS	ICD-10	T436	Poisoning by psychotropic drugs, not elsewhere classified
	OHMRS	DSM-IV	30440	Amphetamine dependence
	OHMRS	DSM-IV	30570	Amphetamine abuse
**Sedatives**	DAD, NACRS	ICD-9	3041	Drug dependence: Sedative, hypnotic or anxiolytic dependence
	DAD, NACRS	ICD-9	9694	Poisoning by Benzodiazepine-based tranquilizers
	DAD, NACRS	ICD-9	3054	Nondependent abuse of drugs: Sedative, hypnotic or anxiolytic abuse
	DAD, NACRS	ICD-10	F13	Mental and behavioural disorders due to use of sedatives or hypnotics
	DAD, NACRS	ICD-10	T423	Poisoning by Barbiturates
	DAD, NACRS	ICD-10	T424	Poisoning by Benzodiazepines
	DAD, NACRS	ICD-10	T425	Poisoning by Mixed antiepileptics, not elsewhere classified
	DAD, NACRS	ICD-10	T426	Poisoning by Benzodiazepines
	DAD, NACRS	ICD-10	T427	Poisoning by Antiepileptic and sedative-hypnotic drugs, unspecified
	DAD, NACRS	ICD-10	T428	Poisoning by Antiparkinsonism drugs and other central muscle-tone depressants
	OHMRS	DSM-IV	30410	Sedative, hypnotic or anxiolytic dependence
	OHMRS	DSM-IV	30540	Sedative, hypnotic or anxiolytic abuse
**Other**	DAD, NACRS	ICD-9	292	Psychoses: Drug-induced mental disorders
	DAD, NACRS	ICD-9	3045	Drug dependence: Hallucinogen dependence
	DAD, NACRS	ICD-9	3046	Drug dependence: Other specified drug dependence
	DAD, NACRS	ICD-9	3048	Combinations of drug dependence excluding opioid type drug
	DAD, NACRS	ICD-9	3049	Drug dependence: Unspecified drug dependence
	DAD, NACRS	ICD-9	3053	Nondependent abuse of drugs: Hallucinogen abuse
	DAD, NACRS	ICD-9	3059	Nondependent abuse of drugs: Other, mixed, or unspecified drug abuse
	DAD, NACRS	ICD-9	6483	Other current conditions in the mother classifiable elsewhere, but complicating pregnancy, childbirth, or the puerperium: drug dependence
	DAD, NACRS	ICD-9	7960	Nonspecific abnormal toxicological findings
	DAD, NACRS	ICD-9	9621	Poisoning by Androgens and anabolic congeners
	DAD, NACRS	ICD-9	9658	Poisoning by Other specified analgesics and antipyretics
	DAD, NACRS	ICD-9	9663	Poisoning by Other and unspecified anticonvulsants including barbituates

Variable	Data source	Code type	Codes	Description
	DAD, NACRS	ICD-9	9664	Poisoning by Anti-Parkinsonism drugs
	DAD, NACRS	ICD-9	9670	Poisoning by Barbiturates
	DAD, NACRS	ICD-9	9684	Poisoning by Other and unspecified general anesthetics
	DAD, NACRS	ICD-9	9685	Poisoning by Surface [topical] and infiltration anesthetics
	DAD, NACRS	ICD-9	9696	Poisoning by Psychodysleptics [hallucinogens]
	DAD, NACRS	ICD-9	9698	Poisoning by Other specified psychotropic agents
	DAD, NACRS	ICD-9	9699	Poisoning by Unspecified psychotropic agent
	DAD, NACRS	ICD-9	970	Poisoning by central nervous system stimulants
	DAD, NACRS	ICD-10	F16	Mental and behavioural disorders due to use of hallucinogens
	DAD, NACRS	ICD-10	F19	Mental and behavioural disorders: multiple drug use and other psychoactive substances
	DAD, NACRS	ICD-10	T387	Poisoning by Androgens and anabolic congeners
	DAD, NACRS	ICD-10	T408	Poisoning by narcotics and psychodysleptics: Lysergide (LSD)
	DAD, NACRS	ICD-10	T409	Poisoning by Other and unspecified psychodysleptics [hallucinogens]
	DAD, NACRS	ICD-10	T412	Poisoning by Other and unspecified general anaesthetics
	DAD, NACRS	ICD-10	T438	Poisoning by Other psychotropic drugs, not elsewhere classified
	DAD, NACRS	ICD-10	T439	Poisoning by Psychotropic drug, unspecified
	DAD, NACRS	ICD-10	T507	Poisoning by Analeptics and opioid receptor antagonists
	DAD, NACRS	ICD-10	Z715	Drug abuse counselling and surveillance
	DAD, NACRS	ICD-10	Z503	Drug rehabilitation
	DAD, NACRS	ICD-10	Z722	Problems related to lifestyle - Drug use
	DAD, NACRS	ICD-10	Z8641	Personal history of psychoactive substance abuse
	OHMRS	DSM-IV	30450	Hallucinogen dependence
	OHMRS	DSM-IV	30480	Polysubstance dependence
	OHMRS	DSM-IV	30490	Other (or unknown) substance dependence
	OHMRS	DSM-IV	30530	Hallucinogen abuse
	OHMRS	DSM-IV	30590	Other (or unknown) substance abuse
**Opioid agonist treatment (OAT)**				
**Physician billing**	OHIP	FEE CODE	K682	OAT monthly management fee, intensive
	OHIP	FEE CODE	K683	OAT monthly management fee, maintenance
	OHIP	FEE CODE	K684	OAT monthly management fee, team premium
**Methadone**	ODB, NMS	PIN	02244290	Metadol-D
	ODB, NMS	PIN	02394596	Methadose

Variable	Data source	Code type	Codes	Description
	ODB, NMS	PIN	02394618	Methadose
	ODB, NMS	PIN	02481979	Methadone HCL concentrate
	ODB, NMS	PIN	02495872	Odan-methadone
	ODB, NMS	PIN	02495880	Odan-methadone
	ODB, NMS	PIN	09850619	Methadone
	ODB, NMS	PIN	09857499	Methadone compounding MMT
	ODB, NMS	PIN	02495783	Methadone HCL
**Buprenorphine/ Naloxone**	ODB, NMS	PIN	02408090	Mylan-buprenorph/naloxone
	ODB, NMS	PIN	02408104	Mylan-buprenorph/naloxone
	ODB, NMS	PIN	02424851	Teva-buprenorphine-naloxone
	ODB, NMS	PIN	02424878	Teva-buprenorphine-naloxone
	ODB, NMS	PIN	02453908	Act buprenorphine/naloxone
	ODB, NMS	PIN	02453916	Act buprenorphine/naloxone
	ODB, NMS	PIN	02502313	Buprenorphine HCL & naloxone HCL
	ODB, NMS	PIN	02502321	Buprenorphine HCL & naloxone HCL
	ODB, NMS	PIN	02502348	Buprenorphine HCL & naloxone HCL
	ODB, NMS	PIN	02502356	Buprenorphine HCL & naloxone HCL
	ODB, NMS	PIN	02502356	Buprenorphine
	ODB, NMS	PIN	02502356	Buprenorphine
	ODB, NMS	PIN	02502356	Buprenorphine
	ODB, NMS	PIN	02468085	Suboxone
	ODB, NMS	PIN	02468093	Suboxone
	ODB, NMS	PIN	02295695	Suboxone
	ODB, NMS	PIN	02295709	Suboxone
**Buprenorphine**	ODB, NMS	PIN	02474921	Probuphine
	ODB, NMS	PIN	02483084	Sublocade
	ODB, NMS	PIN	02483092	Sublocade

Table S5. Codes to identify cirrhosis.

Definition: At least 2 physician visit (OHIP) or at least 1 hospitalization (DAD) or at least 1 ambulatory encounter (any NACRS source) with diagnostic code (excluding suspected diagnosis). NOTE: This is a validated algorithm.

**Code(s)**	**Description**
**ICD-9 codes**	All DXTYPEs are captured from all types of DAD/SDS/NACRS visits (e.g. scheduled admissions, NACRS source = any, ED visits that led to hospitalization, ED visits not seen by physician) unless otherwise specified
4561	ESOPH VARICES W/O BLEED
5712	ALCOHOL CIRRHOSIS LIVER
5715	CIRRHOSIS OF LIVER NOS
**ICD-10-CA codes**	All DXTYPEs are captured from all types of DAD/SDS/NACRS visits (e.g. scheduled admissions, NACRS source = any, ED visits that led to hospitalization, ED visits not seen by physician) unless otherwise specified
I859	Oesophageal varices without bleeding
I982	Oesophageal varices without bleeding in diseases classified elsewhere
I9820	Oesophageal varices in diseases classified elsewhere with bleeding
I9821	Oesophageal varices in diseases classified elsewhere without bleeding
K717	Toxic liver disease with fibrosis and cirrhosis of liver
K703	Alcoholic cirrhosis of liver
K746	Other and unspecified cirrhosis of liver
**OHIP diagnosis codes**	All OHIP non-lab billings are included (e.g., all locations, all billing specialties) unless otherwise specified
571	Cirrhosis of the liver (e.g., alcoholic cirrhosis, biliary cirrhosis)

Table S6. Codes to identify decompensated cirrhosis.

Definition: At least 1 physician visit (OHIP) AND at least 1 hospitalization (DAD) with diagnostic or procedure code (excluding suspected diagnosis). NOTE: This is a validated algorithm.

Code(s)	Description
**ICD-9 codes**	All DXTYPEs are captured from all types of DAD/SDS/NACRS visits (e.g. scheduled admissions, NACRS source = any, ED visits that led to hospitalization, ED visits not seen by physician) unless otherwise specified
4560	ESOPHAG VARICES W BLEED
4562	BLEED ESOPH VAR OTH DIS
45620	BLEED ESOPH VAR OTH DIS
45621	ESOPH VARICE OTH DIS NOS
5722	HEPATIC COMA
5723	PORTAL HYPERTENSION
5724	HEPATORENAL SYNDROME
7824	JAUNDICE NOS
7895	ASCITES
**ICD-10-CA codes**	All DXTYPEs are captured from all types of DAD/SDS/NACRS visits (e.g. scheduled admissions, NACRS source = any, ED visits that led to hospitalization, ED visits not seen by physician) unless otherwise specified
I850	Oesophageal varices with bleeding
I864	Gastric varices
I9820	Oesophageal varices in diseases classified elsewhere with bleeding
I983	Oesophageal varices with bleeding in disease classified elsewhere
K721	Chronic hepatic failure
K729	Hepatic failure, unspecified
K766	Portal hypertension
K767	Hepatorenal syndrome
R17	Unspecified jaundice
R18	Ascites
**CCP codes**	
1006	INSERTION OF SENGSTAKEN TUBE
6691	PERCUTANEOUS ABDOMINAL PARACENTESIS
**CCI codes**	
1NA13BAFA	Control of bleeding, esophagus using endoscopic per orifice approach and banding (varices)
1NA13BAX7	Control of bleeding, esophagus using endoscopic per orifice approach and chemical agent [e.g. ethanolamine, murrhate sodium, polidocanol, sclerosants, tetradecyl sulfate]
1NA13BABD	Control of bleeding, esophagus using endoscopic per orifice approach and balloon (or Sengstaken) tube tamponade
1KQ76GPNR	Bypass, abdominal veins NEC using percutaneous transluminal approach and stent [TIPS] bypass terminating in abdominal vein

1OT52HA	Drainage, abdominal cavity using percutaneous (needle) approach
1OT52HATS	Drainage, abdominal cavity using percutaneous (needle) approach and leaving drainage tube in situ
**OHIP FEE codes**	All OHIP non-lab billings are included (e.g., all locations, all billing specialties) unless otherwise specified
J057	TRANSJUGULAR INTRAHEPATIC PORTOSYSTEMIC SHUNT (TIPS)
Z591	ABD.PERITONEUM,OMENTUM-PARACENTESIS-THERAPEUT.DRAIN. SAMPLE

Table S7. Codes to identify hepatocellular carcinoma (4).

**Inpatient diagnostic codes**	**ICD-9**	**ICD-10**
Malignant neoplasm of liver	155.0	C22.9
Hepatocellular carcinoma		C22.0<br>81703
Combined hepatocellular and cholangiocarcinoma		81803
**Ontario Cancer Registry codes**		
Morphology		Topography
81703 (NOS)<br>81723 (scirrhous)<br>81733 (spindle)<br>81743 (clear cell)<br>81753 (pleomorphic)<br>81803 (Combined hepatocellular carcinoma and cholangiocarcinoma)		C220

Table S8. Codes used to identify liver transplantation.

Definition: At least 1 hospitalization (DAD) OR 1 ambulatory encounter (any NACRS source) with transplant diagnostic or procedure code OR 1 physician billing with OHIP feecode OR transplant receipt in CORR.

**Code(s)**	**Description**
**ICD-9 codes**	All DXTYPEs are captured from all types of DAD/SDS/NACRS visits (e.g. scheduled admissions, NACRS source = any, ED visits that led to hospitalization, ED visits not seen by physician) unless otherwise specified
V427	LIVER TRANSPLANT STATUS
**ICD-10-CA codes**	All DXTYPEs are captured from all types of DAD/SDS/NACRS visits (e.g. scheduled admissions, NACRS source = any, ED visits that led to hospitalization, ED visits not seen by physician) unless otherwise specified
T86400	Liver transplant rejection
T86401	Liver transplant failure
T86402	Liver transplant infection
Z944	Liver transplant status
**CCP codes**	
624	LIVER TRANSPLANT
6240	LIVER TRANSPLANT
6241	AUXILIARY LIVER TRANSPLANT
6249	OTHER TRANSPLANT OF LIVER
**CCI codes**	
1OA85	Transplant, liver
1OA85LAXXK	Transplant, liver of a deceased donor full size liver
1OA85VCXXK	Transplant, liver of a deceased donor multi organ liver with intestine/ pancreas/spleen/stomach [or any combination of]
1OA85WLXXJ	Transplant, liver of a living donor split liver
1OA85WLXXK	Transplant, liver of a deceased donor split liver (or reduced ?paediatric÷ size liver)
**OHIP FEE codes**	All OHIP non-lab billings are included (e.g., all locations, all billing specialties) unless otherwise specified
S294	LIVER-EXC.-LIVER TRANSPLANT-RECIPIENT.
S295	DIGEST.SYST.-LIVER-REPEAT LIVER TRANSPLANT
G254	D&T,PROC.GASTROENTEROLOGY-MGM.POST LIVER TRANSPL'TIMMUNOSUPN
E765	LIVER-RECONST./REPAIR.HEPATIC ARTERY(RE-ANASTOM.CONDUIT)ADD

Table S9. Number of individuals per month initiating HCV treatment, overall and by priority groups.

	**Pre-pandemic 01/01/2015 – 29/02/2020**		**Pandemic 01/03/2020 – 31/12/2022**	
	**N=52,313**		**N=11,378**	
	**Median**	**IQR**	**Median**	**IQR**
**Overall**	323.5	207-383	207.5	186-225
**Sex**				
F	118.0	66-145	75.0	66-81
M	205.5	145-244	131.5	117-145
**Birth cohort**				
<1945	12.0	7-18	3.0	3-4
1945-1964	146.5	119-192	60.0	53-68
1965-1974	63.0	34-77	41.5	35-47
≥1975	96.0	21-121	103.0	90-112
**PWID ever**				
No	145.5	114-188	62.5	54-71
Yes	176.0	81-209	141.0	126-157
**Immigrant status**				
No	292.5	190-344	193.0	174-213
Yes	30.0	20-42	13.0	11-17
**HIV status**				
No	319.0	204-372	205.5	182-220
Yes	8.0	5-11	3.0	2-4
**Homelessness**				
No	310.0	206-375	182.5	162-200
Yes	8.0	4-13	25.0	15-29

HCV: hepatitis C virus; HIV: human immunodeficiency virus; IQR: interquartile range; N: number of observations. PWID: people who inject drugs.

Table S10. Sensitivity Analysis 1 restricting study period to 2018-2022: Treatment initiation rates, the result of interrupted time series analysis.

Model Parameter	Estimate	SE	p-value
**Overall**			
Intercept	51.117	2.62	0.000
Pre-pandemic treatment rate slope, monthly	-1.570	0.18	0.000
Study period (pandemic, ref: pre-pandemic)	-0.696	3.14	0.826
Pandemic treatment rate slope, monthly	-0.104	0.11	0.343
Difference in treatment rate slopes (pandemic *vs.* pre-pandemic)	1.466	0.22	0.000
**Female**			
Intercept	50.082	2.84	0.000
Pre-pandemic treatment rate slope, monthly	-1.614	0.20	0.000
Study period (pandemic, ref: pre-pandemic)	-0.053	3.45	0.988
Pandemic treatment rate slope, monthly	-0.096	0.12	0.419
Difference in treatment rate slopes (pandemic *vs.* pre-pandemic)	1.517	0.23	0.000
**Male**			
Intercept	51.962	2.48	0.000
Pre-pandemic treatment rate slope, monthly	-1.552	0.17	0.000
Study period (pandemic, ref: pre-pandemic)	-1.112	3.00	0.713
Pandemic treatment rate slope, monthly	-0.103	0.10	0.321
Difference in treatment rate slopes (pandemic *vs.* pre-pandemic)	1.449	0.20	0.000
**<1945**			
Intercept	50.983	6.68	0.000
Pre-pandemic treatment rate slope, monthly	-2.070	0.43	0.000
Study period (pandemic, ref: pre-pandemic)	5.628	6.26	0.374
Pandemic treatment rate slope, monthly	-0.080	0.26	0.757
Difference in treatment rate slopes (pandemic *vs.* pre-pandemic)	1.989	0.55	0.001
**1945-1964**			
Intercept	62.650	2.87	0.000
Pre-pandemic treatment rate slope, monthly	-2.066	0.20	0.000
Study period (pandemic, ref: pre-pandemic)	-1.130	3.50	0.750
Pandemic treatment rate slope, monthly	-0.138	0.12	0.250
Difference in treatment rate slopes (pandemic *vs.* pre-pandemic)	1.928	0.24	0.000
**1965-1974**			
Intercept	55.217	2.64	0.000
Pre-pandemic treatment rate slope, monthly	-1.715	0.18	0.000
Study period (pandemic, ref: pre-pandemic)	-0.044	3.28	0.989
Pandemic treatment rate slope, monthly	-0.137	0.11	0.218
Difference in treatment rate slopes (pandemic *vs.* pre-pandemic)	1.578	0.22	0.000
**1975+**			
Intercept	39.931	2.41	0.000
Pre-pandemic treatment rate slope, monthly	-1.072	0.17	0.000
Study period (pandemic, ref: pre-pandemic)	-1.243	3.00	0.680

Model Parameter	Estimate	SE	p-value
Pandemic treatment rate slope, monthly	-0.048	0.10	0.632
Difference in treatment rate slopes (pandemic *vs.* pre-pandemic)	1.024	0.20	0.000
**PWID (ever): YES**			
Intercept	50.836	2.97	0.000
Pre-pandemic treatment rate slope, monthly	-1.399	0.21	0.000
Study period (pandemic, ref: pre-pandemic)	-1.419	3.64	0.698
Pandemic treatment rate slope, monthly	-0.030	0.12	0.808
Difference in treatment rate slopes (pandemic *vs.* pre-pandemic)	1.369	0.24	0.000
**PWID (ever): NO**			
Intercept	52.013	2.40	0.000
Pre-pandemic treatment rate slope, monthly	-1.809	0.17	0.000
Study period (pandemic, ref: pre-pandemic)	0.058	2.94	0.984
Pandemic treatment rate slope, monthly	-0.126	0.10	0.211
Difference in treatment rate slopes (pandemic *vs.* pre-pandemic)	1.683	0.20	0.000
**Immigrant: YES**			
Intercept	44.961	2.35	0.000
Pre-pandemic treatment rate slope, monthly	-1.703	0.16	0.000
Study period (pandemic, ref: pre-pandemic)	2.279	2.71	0.404
Pandemic treatment rate slope, monthly	-0.118	0.09	0.192
Difference in treatment rate slopes (pandemic *vs.* pre-pandemic)	1.585	0.19	0.000
**Immigrant: NO**			
Intercept	53.043	2.79	0.000
Pre-pandemic treatment rate slope, monthly	-1.612	0.19	0.000
Study period (pandemic, ref: pre-pandemic)	-0.638	3.32	0.849
Pandemic treatment rate slope, monthly	-0.091	0.12	0.434
Difference in treatment rate slopes (pandemic *vs.* pre-pandemic)	1.520	0.23	0.000
**HIV: YES**			
Intercept	46.547	3.21	0.000
Pre-pandemic treatment rate slope, monthly	-1.708	0.23	0.000
Study period (pandemic, ref: pre-pandemic)	3.786	4.05	0.354
Pandemic treatment rate slope, monthly	0.057	0.13	0.668
Difference in treatment rate slopes (pandemic *vs.* pre-pandemic)	1.765	0.26	0.000
**HIV: NO**			
Intercept	51.264	2.63	0.000
Pre-pandemic treatment rate slope, monthly	-1.567	0.18	0.000
Study period (pandemic, ref: pre-pandemic)	-0.865	3.16	0.785
Pandemic treatment rate slope, monthly	-0.107	0.11	0.331
Difference in treatment rate slopes (pandemic *vs.* pre-pandemic)	1.459	0.22	0.000
**Homelessness: YES**			
Intercept	24.351	2.83	0.000
Pre-pandemic treatment rate slope, monthly	-0.017	0.19	0.931
Study period (pandemic, ref: pre-pandemic)	-9.799	3.29	0.004

Model Parameter	Estimate	SE	p-value
Pandemic treatment rate slope, monthly	0.247	0.11	0.026
Difference in treatment rate slopes (pandemic *vs*. pre-pandemic)	0.264	0.22	0.239
**Homelessness: NO**			
Intercept	53.168	2.61	0.000
Pre-pandemic treatment rate slope, monthly	-1.692	0.18	0.000
Study period (pandemic, ref: pre-pandemic)	-0.012	3.15	0.997
Pandemic treatment rate slope, monthly	-0.130	0.11	0.237
Difference in treatment rate slopes (pandemic *vs*. pre-pandemic)	1.562	0.22	0.000

HIV: human immunodeficiency virus; PWID: people who inject drugs; ref: reference; SE: standard error.

Table S11. Sensitivity Analysis 1 restricting study period to 2018-2022: Observed vs. counterfactual treatment initiation rates per 1,000 eligible individuals, overall and stratified by priority groups.

**Population**	**Absolute Difference (95% CI)**		
	**May–Dec 2020**	**Jan–Dec 2021**	**Jan–Dec 2022**
**Overall**	8.42 (4.89; 11.96)	23.60 (20.35; 26.84)	37.36 (34.06; 40.66)
**Sex**			
Female	9.40 (5.79; 13.02)	25.75 (22.42; 29.07)	39.81 (36.44; 43.17)
Male	7.58 (3.98; 11.18)	22.67 (19.36; 25.98)	37.06 (33.71; 40.41)
**Birth cohort**			
<1945	15.08 (11.04; 19.11)	36.07 (32.21; 39.92)	58.74 (54.95; 62.53)
1945–1964	11.70 (7.09; 16.32)	31.28 (27.04; 35.52)	49.44 (45.13; 53.75)
1965–1974	10.79 (6.81; 14.77)	26.42 (22.76; 30.08)	42.79 (39.08; 46.50)
≥1975	4.31 (1.73; 6.89)	16.41 (14.03; 18.78)	26.49 (24.10; 28.87)
**PWID status**			
Yes	5.90 (2.47; 9.33)	21.53 (18.37; 24.68)	34.98 (31.80; 38.16)
No	12.51 (8.53; 16.49)	28.34 (24.68; 31.99)	44.29 (40.57; 48.02)
**Immigration status**			
Yes	14.48 (10.66; 18.29)	29.50 (25.99; 33.01)	44.19 (40.61; 47.77)
No	8.22 (4.53; 11.91)	24.27 (20.88; 27.66)	38.77 (35.34; 42.20)
**HIV status**			
HIV (+)	14.21 (9.45; 18.97)	32.83 (28.47; 37.18)	54.69 (50.33; 59.05)
HIV (-)	8.19 (4.67; 11.71)	23.43 (20.19; 26.66)	37.03 (33.75; 40.32)
**Homelessness status**			
Yes	-9.93 (-11.85; -8.01)	-3.61 (-5.36; -1.86)	-3.06 (-4.81; -1.31)
No	10.25 (6.48; 14.01)	25.98 (22.52; 29.43)	40.46 (36.94; 43.98)

CI: Confidence interval; HIV: human immunodeficiency virus; PWID: people who inject drugs.

Table S12. Sensitivity Analysis 2 restricting the treatment-eligible population to individuals with a positive RNA or genotype test: Treatment initiation rates, the result of interrupted time series analysis.

Model Parameter	Estimate	SE	p-value
**Overall**			
Intercept	14.97	2.62	0.0000
Pre-pandemic treatment rate slope, monthly	0.06	0.07	0.3898
Study period (pandemic, ref: pre-pandemic)	-5.20	3.75	0.1697
Pandemic treatment rate slope, monthly	-0.07	0.16	0.6895
Difference in treatment rate slopes during pandemic vs pre-pandemic periods	-0.13	0.19	0.4983
**Female**			
Intercept	13.80	2.74	0.0000
Pre-pandemic treatment rate slope, monthly	0.08	0.07	0.2759
Study period (pandemic, ref: pre-pandemic)	-5.20	3.89	0.1851
Pandemic treatment rate slope, monthly	-0.09	0.17	0.5959
Difference in treatment rate slopes during pandemic vs pre-pandemic periods	-0.17	0.19	0.38
**Male**			
Intercept	15.68	2.55	0.0000
Pre-pandemic treatment rate slope, monthly	0.05	0.07	0.4691
Study period (pandemic, ref: pre-pandemic)	-5.50	3.75	0.1451
Pandemic treatment rate slope, monthly	-0.04	0.16	0.8047
Difference in treatment rate slopes during pandemic vs pre-pandemic periods	-0.09	0.18	0.6216
**<1945**			
Intercept	36.57	4.83	0.0000
Pre-pandemic treatment rate slope, monthly	-0.38	0.13	0.0045
Study period (pandemic, ref: pre-pandemic)	-3.10	7.16	0.6663
Pandemic treatment rate slope, monthly	-0.06	0.30	0.849
Difference in treatment rate slopes during pandemic vs pre-pandemic periods	0.32	0.33	0.3374
**1945-1964**			
Intercept	20.92	3.53	0.0000
Pre-pandemic treatment rate slope, monthly	-0.03	0.10	0.7389
Study period (pandemic, ref: pre-pandemic)	-6.55	4.93	0.1878
Pandemic treatment rate slope, monthly	-0.15	0.22	0.5033
Difference in treatment rate slopes during pandemic vs pre-pandemic periods	-0.11	0.25	0.6479
**1965-1974**			
Intercept	12.66	2.14	0.0000
Pre-pandemic treatment rate slope, monthly	0.13	0.06	0.0263
Study period (pandemic, ref: pre-pandemic)	-7.39	3.31	0.0280
Pandemic treatment rate slope, monthly	-0.04	0.14	0.7844
Difference in treatment rate slopes during pandemic vs pre-pandemic periods	-0.17	0.15	0.2733
**1975+**			
Intercept	3.17	1.40	0.0257

Model Parameter	Estimate	SE	p-value
Pre-pandemic treatment rate slope, monthly	0.26	0.04	0.0000
Study period (pandemic, ref: pre-pandemic)	-5.39	2.10	0.0116
Pandemic treatment rate slope, monthly	0.05	0.09	0.5719
Difference in treatment rate slopes during pandemic vs pre-pandemic periods	-0.21	0.10	0.0354
**PWID (ever): YES**			
Intercept	10.63	1.97	0.0000
Pre-pandemic treatment rate slope, monthly	0.17	0.05	0.0015
Study period (pandemic, ref: pre-pandemic)	-6.33	3.02	0.0385
Pandemic treatment rate slope, monthly	0.06	0.13	0.6170
Difference in treatment rate slopes during pandemic vs pre-pandemic periods	-0.11	0.14	0.4338
**PWID (ever): NO**			
Intercept	18.91	3.33	0.0000
Pre-pandemic treatment rate slope, monthly	-0.04	0.09	0.6167
Study period (pandemic, ref: pre-pandemic)	-5.09	4.56	0.2670
Pandemic treatment rate slope, monthly	-0.16	0.20	0.4464
Difference in treatment rate slopes during pandemic vs pre-pandemic periods	-0.11	0.23	0.6382
**Immigrant: YES**			
Intercept	16.84	3.29	0.0000
Pre-pandemic treatment rate slope, monthly	0.00	0.09	0.9621
Study period (pandemic, ref: pre-pandemic)	-6.21	4.76	0.1947
Pandemic treatment rate slope, monthly	-0.16	0.21	0.4452
Difference in treatment rate slopes during pandemic vs pre-pandemic periods	-0.15	0.23	0.5127
**Immigrant: NO**			
Intercept	14.83	2.53	0.0000
Pre-pandemic treatment rate slope, monthly	0.07	0.07	0.3239
Study period (pandemic, ref: pre-pandemic)	-5.30	3.66	0.1514
Pandemic treatment rate slope, monthly	-0.05	0.16	0.7734
Difference in treatment rate slopes during pandemic vs pre-pandemic periods	-0.11	0.18	0.5297
**HIV: YES**			
Intercept	25.14	3.78	0.0000
Pre-pandemic treatment rate slope, monthly	-0.20	0.10	0.0528
Study period (pandemic, ref: pre-pandemic)	-4.95	5.56	0.3753
Pandemic treatment rate slope, monthly	0.06	0.23	0.8071
Difference in treatment rate slopes during pandemic vs pre-pandemic periods	0.25	0.25	0.3154
**HIV: NO**			
Intercept	14.82	2.60	0.0000
Pre-pandemic treatment rate slope, monthly	0.07	0.07	0.3558
Study period (pandemic, ref: pre-pandemic)	-5.19	3.71	0.1652
Pandemic treatment rate slope, monthly	-0.13	0.18	0.4750
Difference in treatment rate slopes during pandemic vs pre-pandemic periods	-0.07	0.16	0.6782

Model Parameter	Estimate	SE	p-value
**Homelessness: YES**			
Intercept	3.93	2.12	0.0673
Pre-pandemic treatment rate slope, monthly	0.29	0.05	0.0000
Study period (pandemic, ref: pre-pandemic)	-6.39	2.84	0.0271
Pandemic treatment rate slope, monthly	0.43	0.11	0.0003
Difference in treatment rate slopes during pandemic vs pre-pandemic periods	0.14	0.13	0.2579
**Homelessness: NO**			
Intercept	15.38	2.67	0.0000
Pre-pandemic treatment rate slope, monthly	0.05	0.07	0.4820
Study period (pandemic, ref: pre-pandemic)	-5.10	3.82	0.1850
Pandemic treatment rate slope, monthly	-0.09	0.17	0.5732
Difference in treatment rate slopes during pandemic vs pre-pandemic periods	-0.14	0.19	0.4468

HIV: human immunodeficiency virus; PWID: people who inject drugs; ref: reference; SE: standard error.

Table S13. Sensitivity Analysis 2 restricting the treatment-eligible population to individuals with a positive RNA or genotype test: Observed vs. counterfactual treatment initiation rates per 1,000 eligible individuals, overall and stratified by priority groups.

Population	Absolute Difference (95% CI)		
	**May–Dec 2020**	**Jan–Dec 2021**	**Jan–Dec 2022**
**Overall**	-8.8 (-10; -7.6)	-8.2 (-9.2; -7.1)	-9.8 (-10.9; -8.8)
**Sex**			
Female	-9.1 (-10.3; -7.9)	-8.9 (-10; -7.9)	-11.2 (-12.3; -10.2)
Male	-8.7 (-10; -7.3)	-7.7 (-8.9; -6.5)	-9.0 (-10.2; -7.8)
**Birth cohort**			
<1945	-3.1 (-5.6; -0.5)	1.3 (-1.2; 3.7)	5.4 (3; 7.8)
1945–1964	-10.4 (-11.5; -9.3)	-9.6 (-10.6; -8.6)	-11.1 (-12.1; -10.1)
1965–1974	-10.5 (-11.9; -9.0)	-10.3 (-11.6; -9.0)	-13.1 (-14.4; -11.8)
≥1975	-8.3 (-9.9; -6.6)	-8.7 (-10.2; -7.2)	-11.7 (-13.2; -10.2)
**PWID status**			
Yes	-9.4 (-11.1; -7.8)	-8.4 (-9.9; -6.9)	-10.2 (-11.7; -8.8)
No	-8.4 (-9.3; -7.5)	-8.2 (-9; -7.4)	-9.5 (-10.3; -8.7)
**Immigration status**			
Yes	-8.6 (-10.1; -7.1)	-9.4 (-10.7; -8)	-11.6 (-13; -10.3)
No	-8.8 (-10.1; -7.6)	-8.0 (-9.1; -6.8)	-9.5 (-10.7; -8.4)
**HIV status**			
HIV (+)	-4.4 (-6.5; -2.3)	-2.4 (-4.3; -0.5)	2.4 (0.5; 4.3)
HIV (-)	-8.9 (-10.1; -7.7)	-8.2 (-9.3; -7.1)	-10.0 (-11.1; -8.9)
**Homelessness status**			
Yes	-7.9 (-10.5; -5.2)	-2.2 (-4.6; 0.22)	-2.8 (-5.3; -0.4)
No	-8.7 (-9.9; -7.5)	-8.5 (-9.6; -7.3)	-10.3 (-11.4; -9.2)

CI: Confidence interval; HIV: human immunodeficiency virus; PWID: people who inject drugs.

Figure S1. Study flowchart.

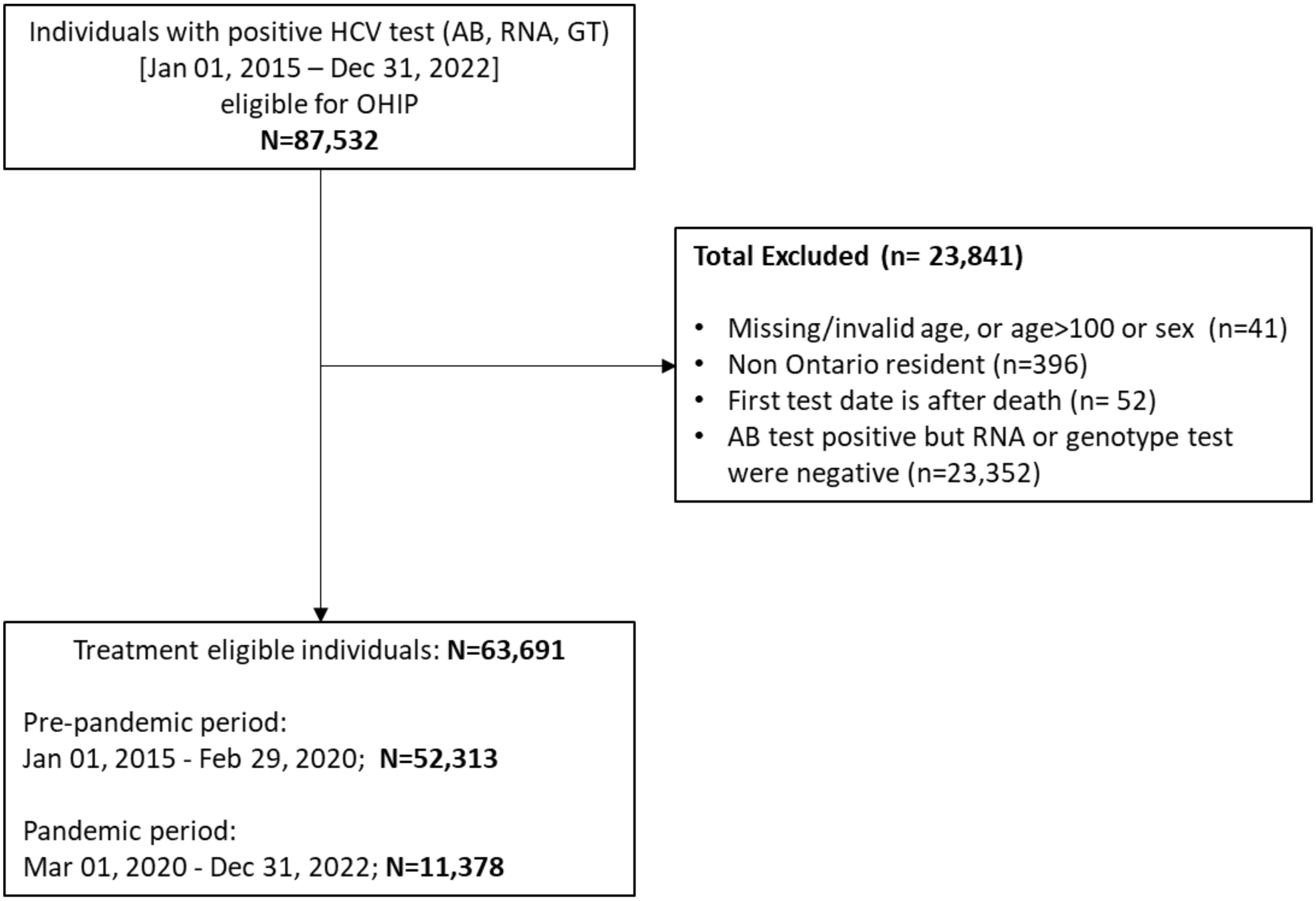

AB: antibody; GT: genotype; RNA: ribonucleic acid.
Note: Individuals eligible for treatment defined as those with a positive RNA or genotype test, as well as those with a positive HCV antibody test without a recorded RNA or genotype result (i.e. unavailable or inconclusive). Individuals who were antibody-positive with negative RNA or genotype testing were excluded.

Figure S2. Number of treatment eligible, treatment initiated individuals and treatment initiation rates based on ODB records for the 2015-2022 and 2018-2022 cohorts.

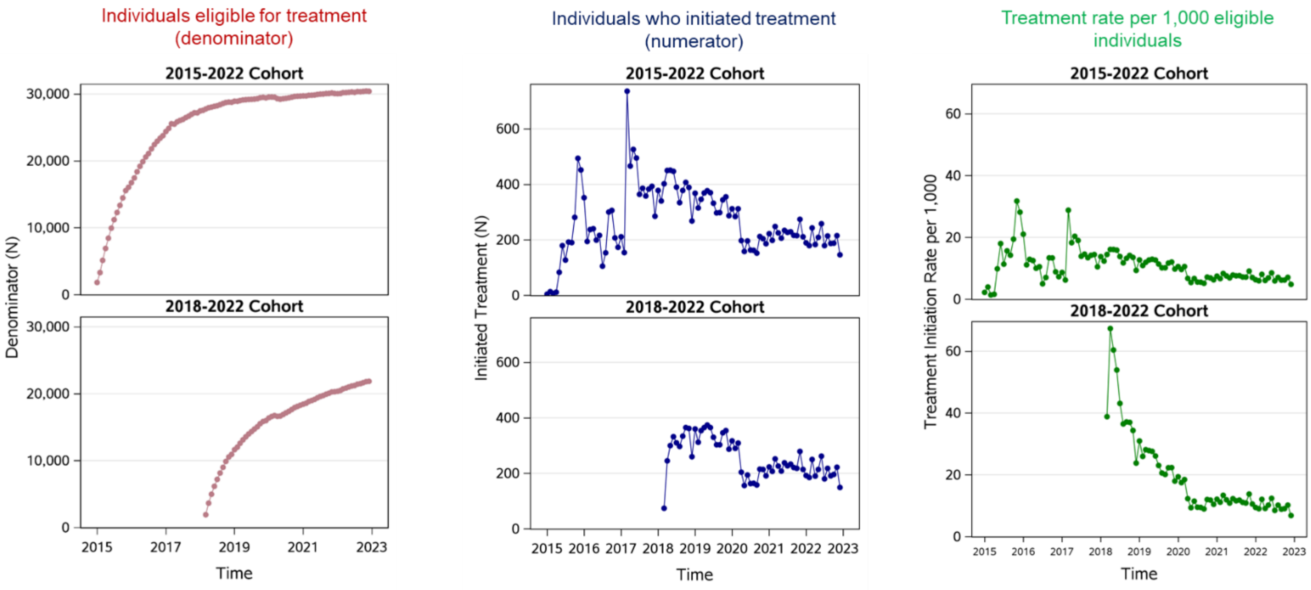

Figure S3. Observed and counterfactual HCV treatment initiation rates by sex.

Monthly rates were calculated per 1,000 eligible individuals, defined as those with a positive RNA or genotype test, as well as those with a positive HCV antibody test without a recorded RNA or genotype result (i.e. unavailable or inconclusive). HCV: hepatitis C virus; RNA: ribonucleic acid.

Female

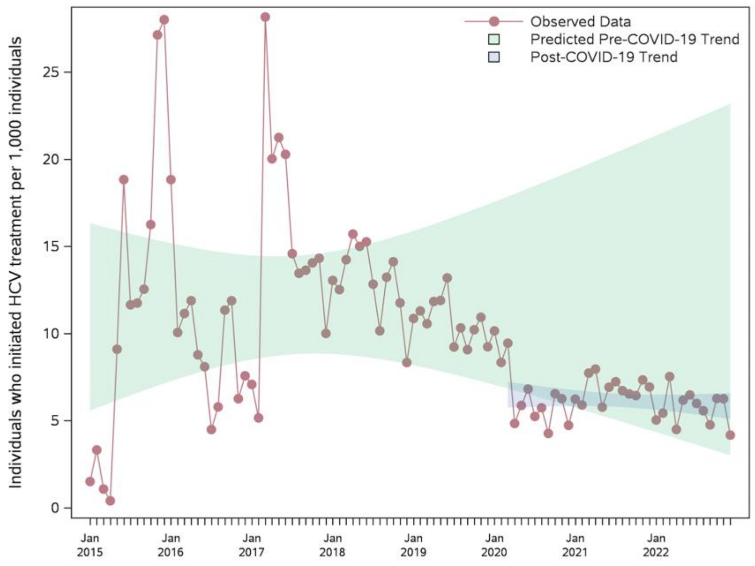

Male

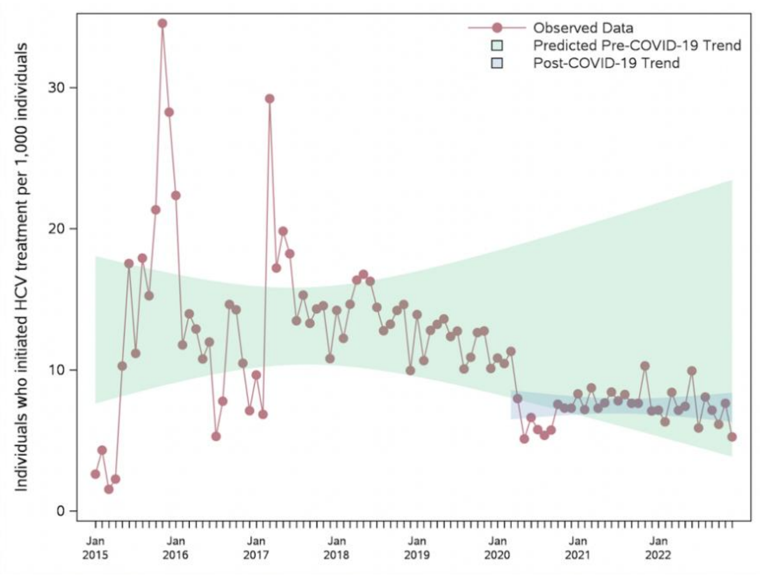

Figure S4. Observed and counterfactual HCV treatment initiation rates by immigration status.

Monthly rates were calculated per 1,000 eligible individuals, defined as those with a positive RNA or genotype test, as well as those with a positive HCV antibody test without a recorded RNA or genotype result (i.e. unavailable or inconclusive). HCV: hepatitis C virus; RNA: ribonucleic acid.

Immigration: YES

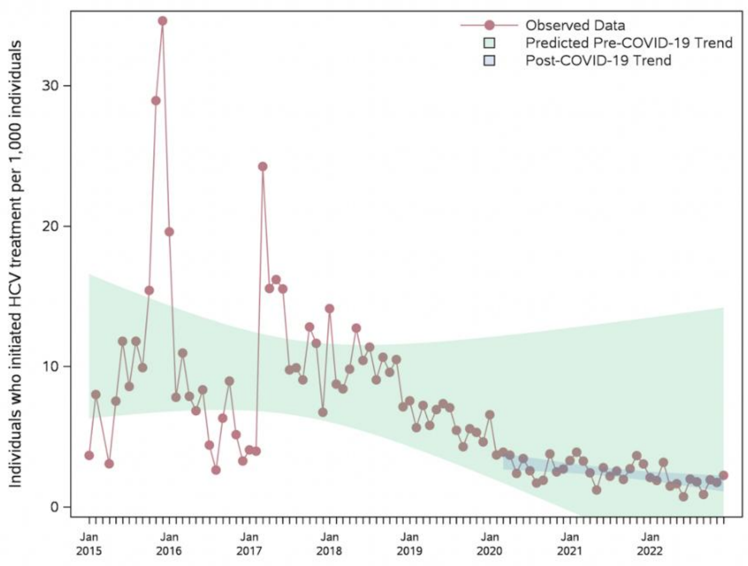

Immigration: NO

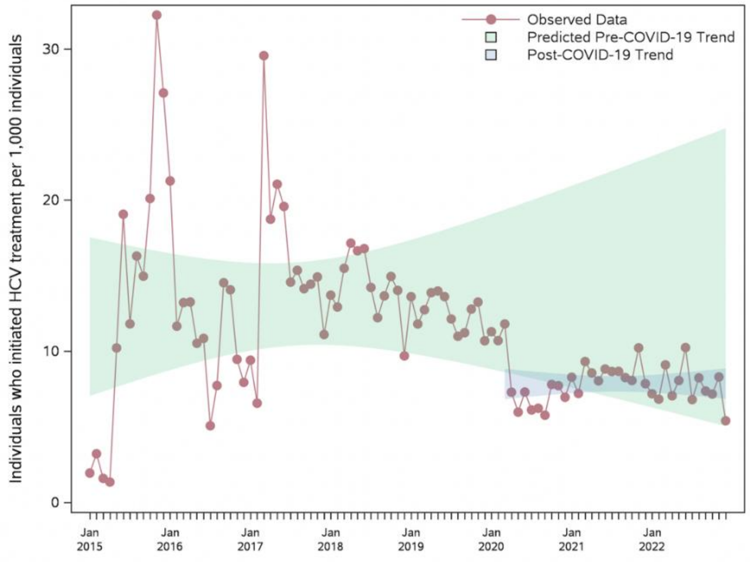

Figure S5. Observed and counterfactual HCV treatment initiation rates by birth cohort.

Monthly rates were calculated per 1,000 eligible individuals, defined as those with a positive RNA or genotype test, as well as those with a positive HCV antibody test without a recorded RNA or genotype result (i.e. unavailable or inconclusive). HCV: hepatitis C virus; RNA: ribonucleic acid.

<1945

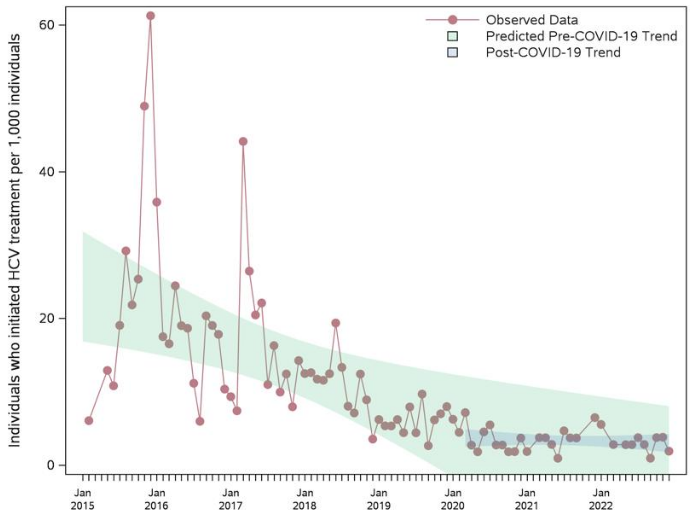

1965-1974

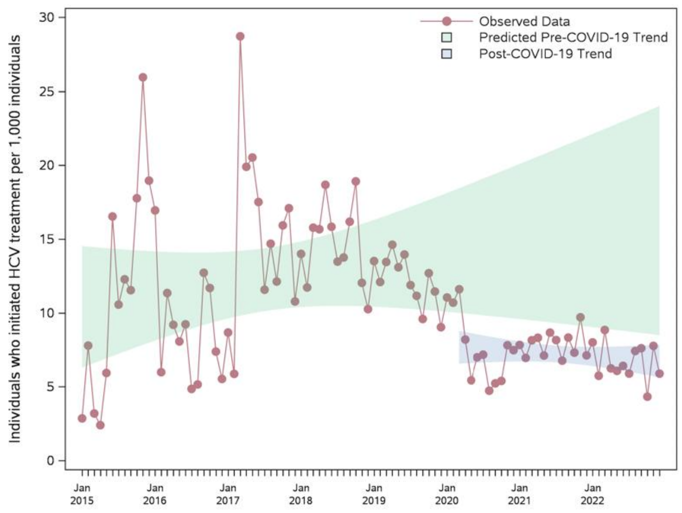

1945-1964

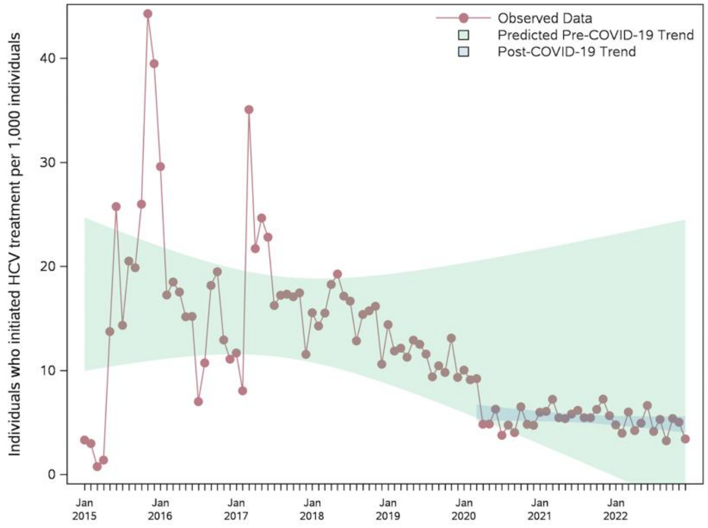

≥1975

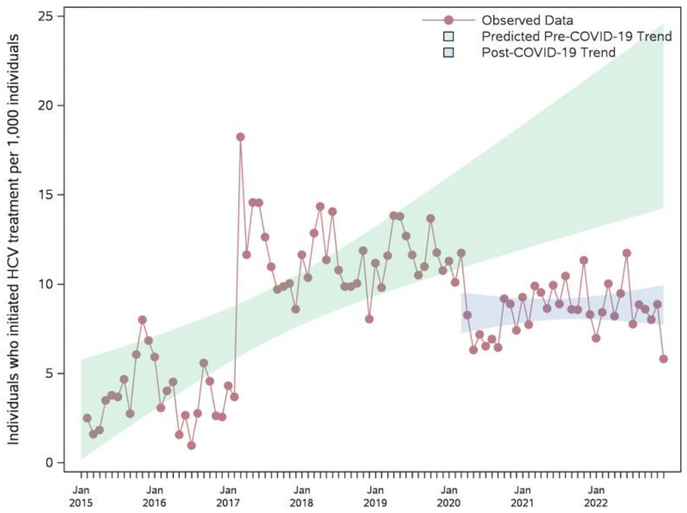
Individuals who initiated HCV treatment per 1,000 individuals
Observed Data
Predicted Pre-COVID-19 Trend
Post-COVID-19 Trend
0
5
10
15
20
25
Jan 2015
Jan 2016
Jan 2017
Jan 2018
Jan 2019
Jan 2020
Jan 2021
Jan 2022

Figure S6. Observed and counterfactual HCV treatment initiation rates by HIV status.

Monthly rates were calculated per 1,000 eligible individuals, defined as those with a positive RNA or genotype test, as well as those with a positive HCV antibody test without a recorded RNA or genotype result (i.e. unavailable or inconclusive). HCV: hepatitis C virus; HIV: human immunodeficiency virus; RNA: ribonucleic acid.

HIV positive

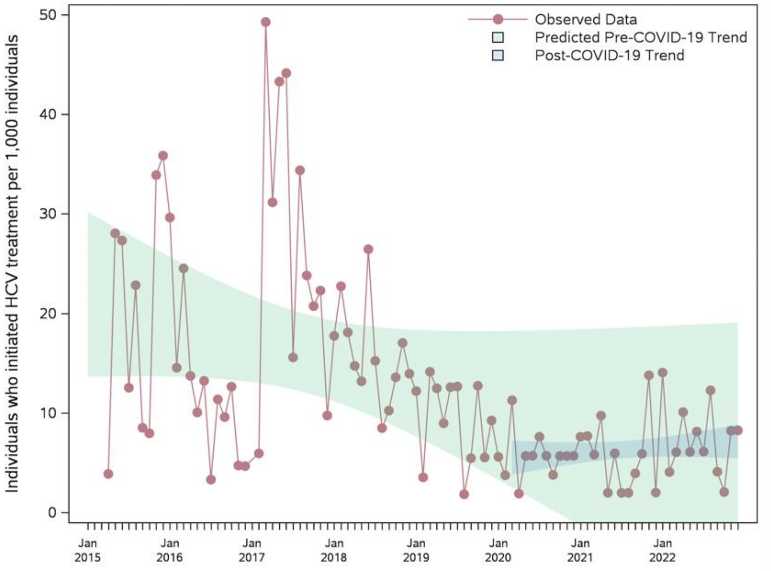

HIV negative

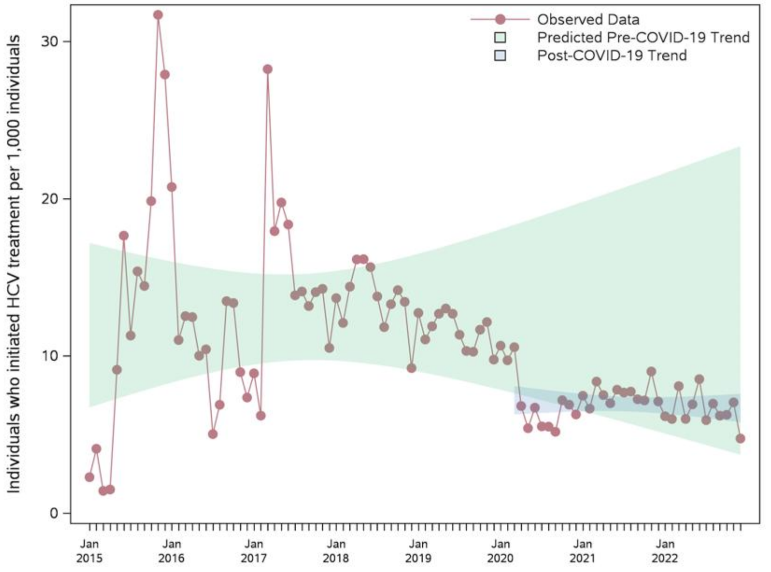

Figure S7. Observed and counterfactual HCV treatment initiation rates by PWID status.

Monthly rates were calculated per 1,000 eligible individuals, defined as those with a positive RNA or genotype test, as well as those with a positive HCV antibody test without a recorded RNA or genotype result (i.e. unavailable or inconclusive). HCV: hepatitis C virus; PWID: people who inject drugs; RNA: ribonucleic acid.

PWID: YES (using high specificity algorithm)

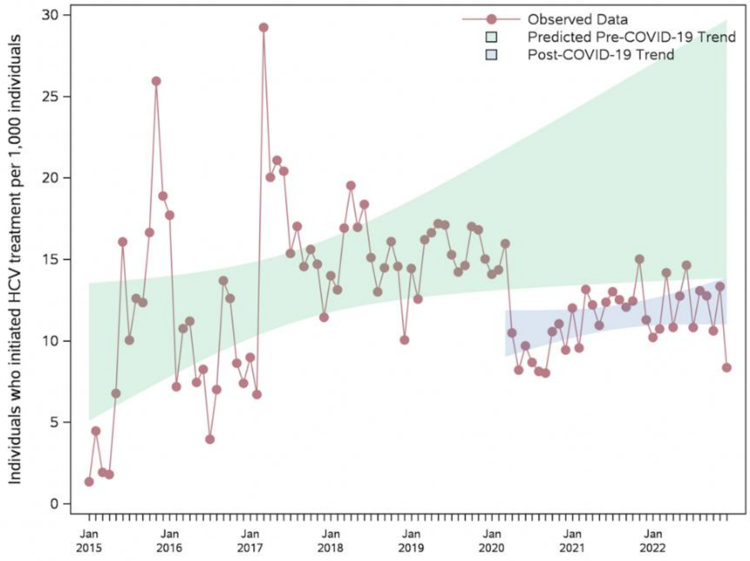

PWID: NO

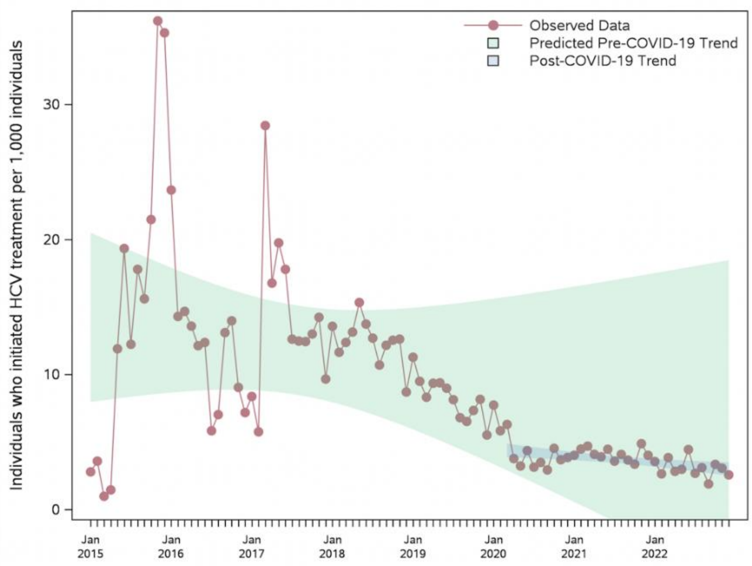

Figure S8. Observed and counterfactual HCV treatment initiation rates by homelessness status.

Monthly rates were calculated per 1,000 eligible individuals, defined as those with a positive RNA or genotype test, as well as those with a positive HCV antibody test without a recorded RNA or genotype result (i.e. unavailable or inconclusive). HCV: hepatitis C virus; RNA: ribonucleic acid.

Homelessness: YES

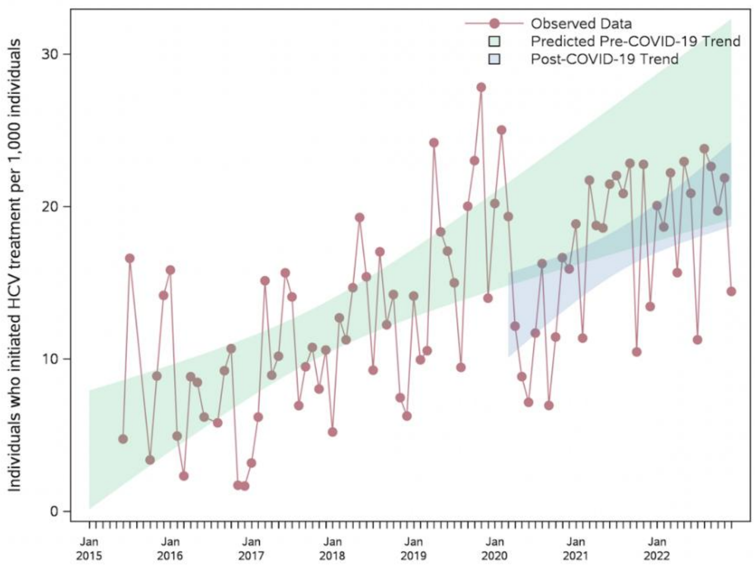

Homelessness: NO

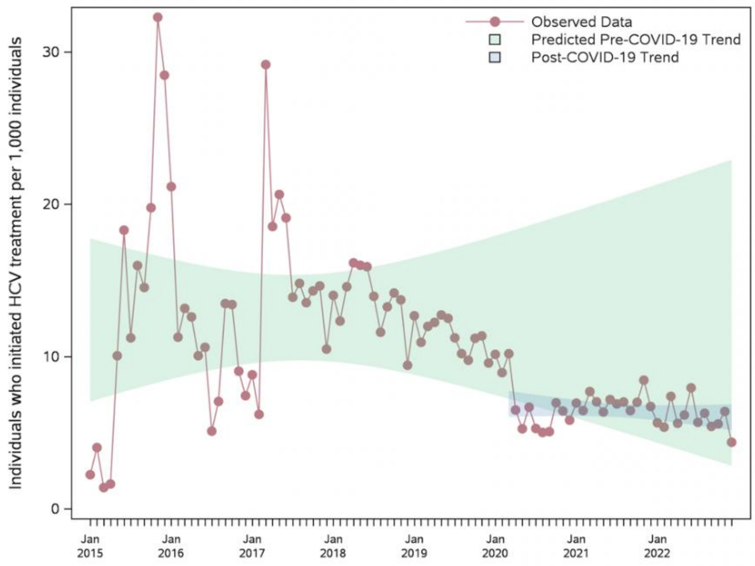

References

1. Richard L, Booth R, Rayner J, Clemens KK, Forchuk C, Shariff SZ. Testing, infection and complication rates of COVID-19 among people with a recent history of homelessness in Ontario, Canada: a retrospective cohort study. CMAJ Open. 2021 Jan-Mar;9(1):E1-E9.
2. Richard L, Carter B, Nisenbaum R, Liu M, Hwang SW. Identification of homelessness using health administrative data in Ontario, Canada following a national coding mandate: a validation study. J Clin Epidemiol. 2024 Aug;172:111430.
3. Greenwald ZR, Werb D, Feld JJ, Austin PC, Fridman D, Bayoumi AM, et al. Validation of case-ascertainment algorithms using health administrative data to identify people who inject drugs in Ontario, Canada. J Clin Epidemiol. 2024 Jun;170:111332.
4. Lapointe-Shaw L, Georgie F, Carlone D, Cerocchi O, Chung H, Dewit Y, et al. Identifying cirrhosis, decompensated cirrhosis and hepatocellular carcinoma in health administrative data: A validation study. PLoS One. 2018;13(8):e0201120.
